# Supplementary material for: SETD7 Expression Is Associated with Breast Cancer Survival Outcomes for Specific Molecular Subtypes: A Systematic Analysis of Publicly Available Datasets
Source: Cancers (Basel). 2022 Dec 7;14(24):6029. doi: 10.3390/cancers14246029 (PMC9775934; doi:10.3390/cancers14246029)
Supplement: Supplementary file 1 [file cancers-14-06029-s001.zip › Supplementary Tables.pdf]

Table S1. Breast cancer datasets with *SETD7* expression (mRNA or protein), mutation or copy number information. HPA – Huma Protein Atlas; MBC – Metastatic Breast Cancer; NA – not available; NGS – Next Generation Sequencing; RPPA – Reverse Phase Protein Arrays; WES – Whole Exome Sequencing.

**Online Tool**	**Type of data** (method)	**Dataset** (information about the sample, n = sample size)	**PAM50 Subtypes** (LumA / LumB / Her2 / Basal / Normal)	**Stage** (0 / 1 / 2 / 3 / 4)	**Grade** (1 / 2 / 3)	**Lymph node status** (positive / negative)	**Therapy** (none / chemotherapy / hormone therapy / both)
**TNMplot**	mRNA (RNA-seq)	TCGA, TARGET, GTEx (paired tumour and adjacent normal tissues, n = 112)	NA	NA	NA	NA	NA
**KM plotter**	mRNA (Gene chip)	E-MTAB-365 (n = 426)	174 / 149 / 53 / 49 / 1	NA	NA	285 / 134	NA
		GSE12276 (tumours with known site of relapse – focus on brain, n = 204)	34 / 72 / 38 / 59 / 1	NA	NA	NA	NA
		GSE16391 (Primary ER+/PR-/HER2- tumours of endocrine-treated patients, includes relapses, n = 48)	27 / 19 / 2 / 0 / 0	NA	2 / 30 / 16	30 / 18	0 / 0 / 30 / 18

		GSE16446 (Primary ER-negative tumours of anthracycline-treated patients (neoadjuvant), n = 107)	0 / 0 / 34 / 72 / 1	NA	2 / 19 / 81	59 / 48	0 / 107 / 0 / 0
		GSE17907 (Tumours with amplification of the *ERBB2* locus, n = 38)	5 / 5 / 24 / 2 / 2	NA	0 / 8 / 29	24 / 11	0 / 23 / 1 / 13
		GSE19615 (n = 115)	45 / 23 / 17 / 30 / 0	NA	23 / 28 / 64	53 / 62	7 / 38 / 21 / 42
		GSE20685 (n = 327)	117 / 91 / 69 / 42 / 8	NA	NA	NA	NA
		GSE20711 (n = 88)	16 / 32 / 17 / 23 / 0	NA	13 / 5 / 70	59 / 29	NA
		GSE21653 (n = 230)	73 / 59 / 21 / 69 / 8	NA	43 / 77 / 107	119 / 108	55 / 43 / 52 / 80
		GSE42568 (naïve tumours, n = 104)	27 / 28 / 12 / 30 / 7	NA	11 / 40 / 53	59 / 45	104 / 0 / 0 / 0
		GSE48390 (breast tumours from Taiwanese women, n = 80)	27 / 28 / 10 / 8 / 7	NA	NA	NA	NA
		GSE58812 (triple-negative (IHC), n = 107)	6 / 4 / 13 / 80 /	NA	NA	NA	NA
		GSE61304 (n = 58)	16 / 15 / 11 / 16 / 0	NA	5 / 16 / 37	37 / 20	NA

		GSE65194 (n = 130)	29 / 17 / 43 / 41 / 0	NA	NA	53 / 58	NA
		GSE9195 (ER+ primary breast tumors of patients treated by Tamoxifen in adjuvant setting, n = 77)	41 / 28 / 7 / 1 / 0	NA	14 / 20 / 24	36 / 41	0 / 0 / 77 / 0
	mRNA (RNA-seq)	GSE96058 (n = 2976)	1504 / 668 / 295 / 309 / 200	NA	449 / 1394 / 1074	1067 / 1820	313 / 340 / 1448 / 854
	Protein (LC-MS/MS)	Tang_2018 (n = 65)	NA	0 / 6 / 46 / 13 / 0	8 / 19 / 28	27 / 37	NA
**HPA**	mRNA (RNA-seq)	TCGA (n = 1075)	NA	0 / 180 / 609 / 243 / 20	NA	NA	NA
**cBioPortal**	Mutation (WES)<br>Copy number (WES)<br>mRNA (RNA-seq)<br>Protein (LC-MS/MS)<br>Phosphoproteome	CPTAC, Cell 2020* (treatment-naive primary tumors, n = 122)	57 / 17 / 14 / 29 / 5	0 / 4 / 70 / 33 / 0 (15 NA)	NA	NA	122 / 0 / 0 / 0

	(LC-MS/MS)						
	Acetylproteome (LC-MS/MS)						
	Mutation (Gene chip)	METABRIC, Nature 2012 & Nat Commun 2016 (n = 2509)	700 / 475 / 224 / 209 / 148 (218 claudin-low / 529 NA / 6 NC)	24 / 630 / 979 / 144 / 11 (721 NA)	214 / 976 / 1198 (121 NA)	1047 / 1196 (266 NA)	543 / 221 / 1025 / 191 (529 NA)
	Copy number (Gene chip)						
	mRNA (Gene chip)						
	Promoter methylation (RRBS)						
	Mutation (WES)	SMC 2018* (tumors from a Korean BC cohort, n = 187)	47 / 65 / 18 / 36 / 2 (19 NA)	0 / 27 / 101 / 58 / 1	NA	NA	(info only on neoadjuvant chemotherapy: no = 178; yes = 9)
	mRNA (RNA-seq)						
	Mutation (WES)	TCGA PanCancer Atlas (n = 1084)	499 / 197 / 78 / 171 / 36 (103 NA)	0 / 277 / 628 / 137 / 39 (3 NA)	NA	687 / 33 (364 NA)	(neoadjuvant therapy: no = 1077; yes = 6; NA = 1)
	Copy number (WES)						
	mRNA (RNA-seq)						
	Protein (RPPA)						
	Mutation (WES)	MBC Project, Provisonal, February 2020*	NA			23 / 92 (65 NA)	(any therapy for more than

	Copy number (WES)	(n = 180)		14 / 44 / 43 / 0 (79 NA)	11 / 27 / 21 / 51 (70 NA)		2 years after diagnosis: no = 131; yes = 46; NA = 3)
	mRNA (RNA-seq)						
	Mutations (NGS)	MSK, Cancer Discovery 2021* (n = 1116)	NA	NA	NA	NA	NA
	Copy number (NGS)						
	Mutations (NGS)	MSK, Cancer Cell 2018 (n = 1756)	NA	556 / 563 / 390 / 395 (14 NA)	98 / 456 / 1175 (189 NA)	765 / 745 (246 NA)	NA
	Copy number (NGS)						
	Mutations (NGS and Sequenom)	MSK, Nature Cancer 2020* (n = 51)	51 / 0 / 0 / 0 / 0	NA	NA	NA	0 / NA / 51 / NA
	Copy number (NGS)						
	Mutations (WES)	MSK, Cancer Res 2020* (n = 32)	NA	NA	NA	NA	NA
	Mutations (NGS)	MSKCC, NPJ Breast Cancer 2019* (n = 68)	68 / 0 / 0 / 0 / 0	NA	NA	NA	NA
	Copy number (NGS)						
	Mutations (targeted	British Columbia, 2012* (n = 65)	0 / 0 / 0 / 38 / 0 (27 NA)	NA	4 / 3 / 56 (2 NA)	27 / 26 (12 NA)	NA

	deep amplicon sequencing)						
	Mutations (WES)	Broad, Nature 2012* (n = 130)	37 / 21 / 18 / 13 / 9 (5 NA)	11 / 73 / 19 / 0	4 / 29 / 26 (44 NA)	NA	NA
	Mutations (WES)	INSERM, PLoS Med 2016* (n = 216)	NA	NA	NA	NA	NA
	Copy number (WES)						
	Mutations (WES)	Sanger, Nature 2012* (n = 100)	NA	NA	8 / 41 / 51	NA	NA

*No survival data

Table S2. Association of *SETD7* expression with therapy, grade and stage using datasets in cBioPortal. Chi-squared test p-value and Benjamini-Hochberg FDR correction q-value. NA – Not available; ND – not determined, either because all samples were treatment-naïve or less than 10% of the patients received therapy; DE – Differentially expressed

Dataset	Analysis (nSETD7 DE / nTotal samples)	Therapy	Grade	Stage
CPTAC - RNA	Overall (61 / 122)	ND	NA	p = 0.24<br>q = 0.35
	Luminal A (29 / 57)	ND	NA	p = 0.38<br>q = 0.57
	Luminal B (9 / 17)	ND	NA	p = 0.45<br>q = 0.94
	Her2-enriched (7 / 14)	ND	NA	p = 0.37<br>q = 0.71
	Basal (15 / 29)	ND	NA	p = 0.95<br>q = 0.95
	Normal-like	-	-	-

	(2 / 5)			
CPTAC - protein	Overall (61 / 122)	ND	NA	p = 0.72<br>q = 0.80
	Luminal A (29 / 57)	ND	NA	p = 0.64<br>q = 0.85
	Luminal B (9 / 17)	ND	NA	p = 0.55<br>q = 0.81
	Her2-enriched (7 / 14)	ND	NA	p = 0.65<br>q = 0.86
	Basal (15 / 29)	ND	NA	p = 0.45<br>q = 0.70
	Normal-like (2 / 5)	-	-	-
METABRIC	Overall (952 / 2509)	Hormone therapy:<br>p = 0.13<br>q = 0.45	p = 0.92<br>q = 0.95	p = 0.05<br>q = 0.31

		Chemotherapy:<br>p = 0.35<br>q = 0.62		
	luminal A<br>(339 / 700)	Hormone therapy:<br>p = 0.88<br>q = 0.99	p = 0.30<br>q = 0.99	p = 0.72<br>q = 0.99
	luminal B<br>(231 / 475)	Hormone therapy:<br>p = 0.90<br>q = 0.98	p = 0.36<br>q = 0.98	p = 0.13<br>q = 0.98
	Her2-enriched<br>(110 / 224)	Hormone therapy:<br>p = 0.08<br>q = 0.89<br><br>Chemotherapy:<br>p = 0.33	p = 0.14<br>q = 0.89	p = 0.09<br>q = 0.89

		q = 0.89		
	Basal (99 / 209)	Hormone therapy: p = 0.61 q = 0.95<br><br>Chemotherapy: p = 0.37 q = 0.95	p = 0.54 q = 0.95	p = 0.92 q = 0.97
	Normal-like (70 / 148)	Hormone therapy: p = 0.63 q = 1.00<br><br>Chemotherapy: p = 0.78 q = 1.00	p = 0.86 q = 1.00	p = 0.07 q = 0.79

	Caludin-low (99 / 218)	Hormone therapy: p = 0.92 q = 0.98<br><br>Chemotherapy: p = 0.95 q = 0.98	p = 0.17 q = 0.89	p = 0.18 q = 0.89
SMC	Overall (84 / 187)	ND	NA	p = 0.67 q = 0.71
	Luminal A (23 / 47)	ND	NA	p = 0.44 q = 0.97
	Luminal B (33 / 65)	ND	NA	p = 0.64 q = 0.76
	Her2-enriched (9 / 18)	ND	NA	p = 0.29 q = 0.60
	Basal (18 / 36)	ND	NA	p = 0.12 q = 0.43

	Normal-like<br>(- / 2)	-	-	-
TCGA PanCancer Atlas	Overall<br>(541 / 1084)	ND	NA	p = 0.10<br>q = 0.19
	Luminal A<br>(249 / 499)	ND	NA	p = 0.42<br>q = 0.62
	Luminal B<br>(99 / 197)	ND	NA	p = 0.46<br>q = 0.95
	Her2-enriched<br>(39 / 78)	ND	NA	p = 0.24<br>q = 0.81
	Basal<br>(85 / 171)	ND	NA	p = 0.56<br>q = 0.88
	Normal-like<br>(18 / 36)	ND	NA	p = 0.47<br>q = 0.69
MBC Project	Overall<br>(73 / 180)	Any:<br>p = 0.62<br>q = 0.91	p = 0.69<br>q = 0.97	p = 0.47<br>q = 0.88
	ER+	Any:	p = 0.23	p = 0.38

	(33 / 113)	p = 0.36<br>q = 0.93	q = 0.93	q = 0.93
	ER-<br>(7 / 23)	Any:<br>p = 0.91<br>q = 0.94	p = 0.33<br>q = 0.94	p = 0.29<br>q = 0.94
	PR+<br>(25 / 85)	Any:<br>p = 0.25<br>q = 0.91	p = 0.15<br>q = 0.91	p = 0.13<br>q = 0.91
	PR-<br>(14 / 43)	Any:<br>p = 0.88<br>q = 0.99	p = 0.39<br>q = 0.99	p = 0.35<br>q = 0.99
	HER2+<br>(11 / 37)	Any:<br>p = 0.89<br>q = 0.99	p = 0.91<br>q = 0.99	p = 0.42<br>q = 0.83
	HER2-<br>(28 / 85)	Any:<br>p = 0.11<br>q = 0.98	p = 0.92<br>q = 0.98	p = 0.42<br>q = 0.98

Table S3. Association of *SETD7* differentially expression and known SETD7 targets and their methylation sites and other sites known to compete with SETD7 methylation. Significant associations are highlighted in bold (Benjamini-Hochberg q-val ≤ 0.05 and |logFC|<0.4). NA – not available; NS – not significant.

**Protein (gene)**	**Methylation site**	**Competing processes** (sites)	**TCGA PanCancer** (RPPA)		**CPTAC- protein** (LC -MS/MS)		
			**Overall** (n = 541 / 1084)	**Luminal A** (n = 249 /499)	**Overall** (n = 60 / 122)		
					**Acetylproteome**	**Phosphoproteome**	**Total Proteome**
AKAP-6 (*AKAP6*)	K604 [1]	-	NA	NA	NA	NS (AKAP6_S1007s)	NA
Androgen Receptor (*AR*)	K630 [2] and K632 [3]	-	**High SETD7 (AR)**	NS (AR)	NA	**High SETD7 (AR_S310s, AR_S651s)** NS (AR_S516s, AR_S96s)	**High SETD7**
Catenin beta-1 (*CTNNB1*)	K180 [4]	-	NS* (CTNNB1)	NS* (CTNNB1)	NA	**Low SETD7 (CTNNB1_S191s)**	NS

						NS (CTNNB1_S179s, CTNNB1_S389s_S 425s_T428t, CTNNB1_T42t, CTNNB1_S552s, CTNNB1_S675s)	
Centromere protein C (*CENPC* / *CENPC1*)	K414 [1]	-	NA	NA	NA	**Low SETD7 (CENPC_S177s, CENPC_S225s, CENPC_S538s, CENPC_S709s_S7 10s, CENPC_S73s, CENPC_S773s, CENPC_T130t, CENPC_T734t)** NS (CENPC_S528s, CENPC_S763s_S77	**Low SETD7**

						3s, CENPC_S384s, CENPC_S709s_S710s_S713s, CENPC_S620s, CENPC_S333s, CENPC_S316s, CENPC_S276s, CENPC_S709s)	
Cullin 1 (*CUL1*)	K73 [1]	-	NA	NA	NA	NA	**Low SETD7**
Dnmt1 (*DNMT1*)	K142 [5]	-	NA	NA	NS (DNMT1_K189k, DNMT1_K973k)	**Low SETD7 (DNMT1_S1105s, DNMT1_S127s, DNMT1_S154s, DNMT1_S398s, DNMT1_S714s, DNMT1_S732s, DNMT1_T253t)**	**Low SETD7**

						NS (DNMT1_T328t, DNMT1_S6s, DNMT1_S143s, DNMT1_S141s_S143s, DNMT1_T166t, DNMT1_S1447s, DNMT1_S312s)	
ERα (*ESR1*)	K302 [6]	-	**High SETD7 (ESR1)** NS (ESR1_PS118)	**High SETD7 (ESR1)** NS* (ESR1_PS118)	NS (ESR1_K171k)	**High SETD7 (ESR1_S296s)** NS (ESR1_S167s)	NS
E2F-1 (*E2F1*)	K185 [7]	Phosphorylation (S31 and S364)	NA	NA	NA	NS (E2F1_S375s, E2F1_S307s)	NA

		Acetylation (K117, K120 and K125) [8]					
Forkhead box protein O3 (*FOXO3*)	K271 [9,10]	-	NS* (FOXO3) NS (FOXO3_PS318_S321)		NS (FOXO3_K259k, FOXO3_K242k)	NS (FOXO3_S555s, FOXO3_S253s, FOXO3_S413s, FOXO3_T433t, FOXO3_S55s, FOXO3_S294s_S300s, FOXO3_S284s, FOXO3_S311s, FOXO3_S294s, FOXO3_T32t, FOXO3_T433t_S439s, FOXO3_S215s, FOXO3_S280s,	NS

						FOXO3_T427t, FOXO3_S300s)	
Transcriptional activator GLI3 (*GLI3*)	K436 and K595 [11]	-	NA	NA	NA	**High SETD7 (GLI3_S1006s, GLI3_S445s)** NS (GLI3_S980s, GLI3_S849s, GLI3_S664s, GLI3_S313s, GLI3_S907s, GLI3_S865s, GLI3_S877s, GLI3_S1038s)	**High SETD7**
Hif1-alpha (*HIF1A*)	K32 [12]	-	NA	NA	NS (HIF1A_K733k)	NS (HIF1A_S475s)	NA
HIV Tat-specific factor 1	K51 [13] and K71 [14]	-	NA	NA	NS (HTATSF1_K661k)	**Low SETD7 (HTATSF1_S702s, HTATSF1_S713s,**	**Low SETD7**

(*HTATSF1*)						**HTATSF1_S521s, HTATSF1_S676s, HTATSF1_S494s, HTATSF1_S616s_ S624s, HTATSF1_S494s_ S498s, HTATSF1_S521s_ S529s, HTATSF1_S713s_ S714s, HTATSF1_S721s, HTATSF1_S529s, HTATSF1_S481s, HTATSF1_S616s, HTATSF1_S600s)** NS (HTATSF1_S624s,	

						HTATSF1_S597s_S600s, HTATSF1_S407s, HTATSF1_T633t, HTATSF1_S498s, HTATSF1_S387s, HTATSF1_S445s_S452s_S453s, HTATSF1_S85s_S498s, HTATSF1_S713s_S714s_S721s, HTATSF1_S561s, HTATSF1_S479s, HTATSF1_S485s, HTATSF1_S453s, HTATSF1_S452s_S453s,	

						HTATSF1_S544s, HTATSF1_S241s, HTATSF1_S616s_S624s_T633t, HTATSF1_S403s_S407s, HTATSF1_S642s, HTATSF1_S579s, HTATSF1_S387s_S389s, HTATSF1_S445s, HTATSF1_S702s_S713s, HTATSF1_359s, HTATSF1_S597s_S600s_S607s, HTATSF1_S403s)	
IRF-1 (*IRF1*)	K126 [1]	-	NA	NA	NA	NA	NA

Histone acetyltransferase KAT2B (*KAT2B* / *PCAF*)	K78, K89, K638, K671, K672 and K692 [15]	-	NA	NA	NA	NA	NA
KH domain-containing, RNA-binding, signal transduction-associated protein 1, Sam68 (*KHDRBS1*)	K208 [16]	-	NA	NA	**Low SETD7 (KHDRBS1_K169k, KHDRBS1_K175k)** NS (KHDRBS1_K152k, KHDRBS1_K165k, KHDRBS1_K139k, KHDRBS1_K194k)	NS (KHDRBS1_S20s, KHDRBS1_S35s, KHDRBS1_S58s, KHDRBS1_S137s, KHDRBS1_T33t, KHDRBS1_T61t, KHDRBS1_S90s, KHDRBS1_T84t)	NS
Protein lin-28 homolog A (*LIN28A*)	K135 [17]	-	NA	NA	NA	NA	NA

MeCp2 (*MECP2*)	K347 [1]	-	NA	NA	NS (MECP2_K222k, MECP2_K461k, MECP2_K333k, MECP2_K231k MECP2_K343k, MECP2_K316k, MECP2_K34k)	**High SETD7 (MECP2_S286s, MECP2_T323t, MECP2_T323t_S325s)** NS (MECP2_S325s, MECP2_T240s, MECP2_S92s, MECP2_S176s, MECP2_S25s, MECP2_S435s, MECP2_S82s, MECP2_S372s, MECP2_413s, MECP2_161s, MECP2_S368s_S372s)	NS

Mediator of RNA polymerase II transcription subunit 1 (*MED1* / *PPARBP*)	K1006 [1]	-	NA	NA	**Low SETD7 (MED1_K699k)** NS (MED1_K2k, MED1_K1210k, MED1_K1529k, MED1_K1309k, MED1_K1126k, MED1_K994k, MED1_K1354k, MED1_K991k)	**Low SETD7 (MED1_S1207s, MED1_S1207s_T1215t)** NS (MED1_S1481s_S1482s, MED1_S1463s, MED1_T1215t, MED1_S1453s_S1463s, MED1_S1479s, MED1_T1051t, MED1_S1463s_S1465s, MED1_S588s, MED1_S1302s, MED1_S1192s, MED1_S1251, MED1_T628t,	

						MED1_S1433s_T1440t, MED1_S1479s_S1481s_S1482S, MED1_S1156s, MED1_S953s, MED1_S1437s, MED1_T1017t_T1032t, MED1_S1130s, MED1_S664s, MED1_T1051t_T1057t, MED1_S1433s, MED1_S1401s, MED1_S1023s)	
Bile acid receptor (*NR1H4* / *FXR*)	K206 [18]	-	NA	NA	NA	NA	NA

PARP-1 (*PARP1*)	K508 [19]	-	NA	NA	**Low SETD7 (PARP1_K621k, PARP1_K400k, PARP1_K662k, PARP1_K108k, PARP1_K633k, PARP1_K418k, PARP1_K105k, PARP1_K134k)** NS (PARP1_K700k, PARP1_K637k, PARP1_K796k, PARP1_K600k, PARP1_K433k, PARP1_K683k, PARP1_K654k, PARP1_K221k, PARP1_K84k,	**Low SETD7 (PARP1_S179s, PARP1_S782s, PARP1_T368t)** NS (PARP1_S519s, PARP1_S257s)	**Low SETD7**

					PARP1_K518k, PARP1_K119k, PARP1_K521k, PARP1_K505k, PARP1_K148k, PARP1_K192k, PARP1_K548k)		
PDX-1 (*PDX1*)	K123 and K131 [20]	-	NA	NA	NA	NA	NA
PGC-1-alpha (*PPARGC1A*)	K779 [21]	-	NA	NA	NA	NA	NA
Protein phosphatase 1 regulatory subunit 12A (*PPP1R12A*)	K442 [22]	-	NA	NA	NA	**Low SETD7 (PPP1R12A_S422s) High SETD7 (PPP1R12A_S427s _S473s, PPP1R12A_S479s, PPP1R12A_S507s,**	**High SETD7**

						**PPP1R12A_S618s, PPP1R12A_S554s, PPP1R12A_S668s, PPP1R12A_S695s, PPP1R12A_S862s, PPP1R12A_S862s_ S871s, PPP1R12A_S910s, PPP1R12A_T443t)** NS (PPP1R12A_S445s, PPP1R12A_S292s, PPP1R12A_S995s, PPP1R12A_S903s, PPP1R12A_T853, PPP1R12A_S477s, PPP1R12A_S409s, PPP1R12A_S507s_	

						T508t, PPP1R12A_T524st, PPP1R12A_T853t_ S682s_S871s, PPP1R12A_S509s, PPP1R12A_S608s, PPP1R12A_S299s, PPP1R12A_S527s, PPP1R12A_T406t_ S409s, PPP1R12A_S356s_ S357s_S365s, PPP1R12A_T305t)	
pRb (*RB1*)	K810 [23] ad K873 [24]	Phosphorylation (S807 and S811) [23]	NS* (RB1_PS807_ S811)	NS (RB1_PS807_ S811)	NA	**High SETD7 (RB1_T601t)** NS (RB1_T841t, RB1_Y805yS807s, RB1_S780s_S788s,	

						RB1_T356t, RB1_S37s, RB1_S807s, RB1_S249s, RB1_T373t, RB1_T601t_S612s, RB1_S780s, RB1_S795s, RB1_T821t_T826t, RB1_T826t, RB1_T821t, RB1_S249s_T252t)	
Transcription factor p65, NFkB-p65 (*RELA*)	K37 [25], K315 and K316 [26]	-	NA	NA	NS (RELA_K122k, RELA_K195k, RELA_K310k)	**High SETD7 (RELA_S205s)** NS (RELA_S238s, RELA_S338s, RELA_S45s)	NS

Serine/threonine-protein kinase RIO1 (*RIOK1*)	K411 [27]	Phosphorylation (T410) [27]	NA	NA	NA	**Low SETD7 (RIOK1_S21s_S22s, RIOK1_S22s)** NS (RIOK1_S21s_S22s_S24s)	**Low SETD7**
Nuclear receptor ROR-alpha (*RORA*)	K87 [28]	-	NA	NA	NA	NS (RORA_S49s)	NA
60S ribosomal protein L29 (*RPL29*)	K5 [29]	-	NA	NA	NA	NS (RPL29_S142s, RPL29_S158s)	NS
hSIRT1 (*SIRT1*)	K233, K235, K236 and K238 [30]	-	NA	NA	NA	NS (SIRT1_T719t, SIRT1_S47s, SIRT1_S27s, SIRT1_S14s, SIRT1_747s,	NS

						SIRT1_S615s, SIRT1_S14s_S16s)	
Mothers against decapentaplegic homolog 7 (*SMAD7*)	K70 [31]	-	NA	NA	NA	NA	NA
Transcription factor SOX-2 (*SOX2*)	K119 [32]	-	NA	NA	NA	NA	NA
Msx2-interacting protein (*SPEN* / *MINT*)	K2076 [1]	-	NA	NA	NS (SPEN_K21k)	**High SETD7 (SPEN_S1425s) Low SETD7 (SPEN_S1636s, SPEN_S2114s_S2126s, SPEN_S2120s, SPEN_S2126s, SPEN_S736s_S740**	NS

						**s, SPEN_S749s, SPEN_T1633t_S1636s, SPEN_T2393t, SPEN_T2421t)** NS (SPEN_S2412s, SPEN_T1910t, SPEN_1619t_S1622s, SPEN_S1252s, SPEN_S1222s, SPEN_T3467t, SPEN_T3139t, SPEN_S1380s_S1382s, SPEN_S1918s, SPEN_T1619t, SPEN_T1947t, SPEN_S2366s, SPEN_T1140t, SPEN_S725s_S727s	

						, SPEN_S727s, SPEN_S1857, SPEN_S1287s, SPEN_S1283s_S1287s, SPEN_S623s, SPEN_S3433s, SPEN_S1278s, SPEN_S2101s, SPEN_S2466s, SPEN_S190s_2, SPEN_S2159s, SPEN_S1268s, SPEN_S190s_1, SPEN_S736s, SPEN_S1261s, SPEN_T3236t, SPEN_S2493s, SPEN_T2918t,	

						SPEN_S1268s_S1278s, SPEN_S1062s, SPEN_S1380s, SPEN_T1826t, SPEN_S1006s)	
Signal transducer and activator of transcription 3 (*STAT3*)	K140 [33]	-	NS (STAT3_PY705)	NS (STAT3_PY705)	**Low SETD7 (STAT3_K631k)** NS (STAT3_K370k, STAT3_K383k)	NA	NS
Histone-lysine N-methyltransferase SUV39H1 (*SUV39H1*)	K105 and K123 [34]	-	NA	NA	NA	**Low SETD7 (SUV39H1_S402s)**	NS

Transcription initiation factor TFIID subunit 7 (*TAF7*)	K5 [35]	-	NA	NA	NS (TAF7_K291k)	**Low SETD7 (TAF7_T274t)** NS (TAF7_S201s, TAF7_S171s, TAF7_S213s, TAF7_S264s, TAF7_S159s)	NS
Transcription initiation factor TFIID subunit 10 (*TAF10*)	K189 [36]	-	NA	NA	NA	NS (TAF10_S44s)	NS*
Cellular tumor antigen p53 (*TP53*)	K372 [37]	-	NS* (TP53)	NS	NS (TP53_K381k_K382k)	NS (TP53_S315s, TP53_S367s, TP53_S15s, TP53_S392s)	NS
Dual specificity	K708 [1]	-	NA	NA	NA	NA	**Low SETD7**

protein kinase TTK (*TTK* / *MPS1*)							
Transcriptional coactivator YAP1 (*YAP1*)	K494 [38]	-	NS* (YAP1, YAP1_PS127)	NS (YAP1)<br>NS* (YAP1_PS127)	NS (YAP1_K97k, YAP1_K346k)	**High SETD7 (YAP1_S109s_T110t, YAP1_S128s, YAP1_S164s, YAP1_S386s, YAP1_T365t)**<br>NS (YAP1_S109s, YAP1_S131_T143t, YAP1_S289s, YAP1_T432t, YAP1_S274s, YAP1_S61s, YAP1_S276s, YAP1_S217s,	**High SETD7**

						YAP1_S229s, YAP1_S344s)	
Transcriptional repressor protein YY1 (*YY1*)	K173 and K411 [39]	-	NA	NA	**Low SETD7 (YY1_K339k)** NS (YY1_K351k)	**Low SETD7 (YY1_S118s)** NS (YY1_S187s)	NS
Transcription factor YY2 (*YY2*)	247 [40]	-	NA	NA	NA	NA	NA
Palmitoyltransferase ZDHHC8 (*ZDHHC8*)	K300 [1]	-	NA	NA	NA	NS (ZDHHC8_S606s, ZDHHC8_S526s, ZDHHC8_S453s, ZDHHC8_S675s)	NA

* not significant because |logFC|<0.4

Table S4. SETD7 association with survival outcomes. Significant values are highlighted in bold and strong tendencies (p-val ≤ 0.1) are shown in italic. Log rank test. BC – Breast Cancer, CI – Confidence Interval, DE – Differential Expression, DFS – Disease Free Survival, DSFS – Disease-Specific Free Survival, HPA – Huma Protein Atlas, HR – Hazard Ratio, OS – Overall survival, PFS – Progression Free Survival, PPS – Palliative Performance Score, RFS – Recurrence Free Survival, DMFS – Distant Metastasis Free Survival.

**Online tool**	**Type of data**	**Dataset** (additional information)	**PAM50 Subtypes** (lumA / lumB / Her2-enriched / basal / normal-like)	**nSETD7 DE / nTotal samples**	**Survival**	**p-value**	**HR (CI) / Prognosis associated with high SETD7**
**KM plotter**	Gene chip	All (E-MTAB-365 and 13 GEO studies)	631 / 566 / 358 / 442 / 35	1015 / 2032	RFS	0.96	1.01 (0.81 – 1.25)
		All (7 GEO studies)	259 / 183 / 218 / 283 / 15	482 / 958	DMFS	0.25	0.79 (0.54 – 1.18
		All (7 GEO studies)	222 / 200 / 198 / 296 /27	473 / 943	OS	*0.066*	*0.69 (0.46 – 1.03) Good*
		All (3 GEO studies)	36 / 53 / 47 / 38 / 6	90 / 108	PPS	**0.007**	**0.49 (0.29 – 0.83) Good**
		E-MTAB-365	174 / 149 / 53 / 49 / 1	212 / 426	RFS	0.57	0.86 (0.52 – 1.43)

		GSE12276 (tumours with known site of relapse – focus on brain)	34 / 72 / 38 / 59 / 1	104 / 204	RFS	0.13	0.74 (0.5 – 1.09)
		GSE16391 (Primary ER+/PR-/HER2- tumours of endocrine-treated patients, includes relapses)	27 / 19 / 2 / 0 / 0	24 / 48	RFS	*0.1*	*5.04 (0.59 – 43.35) Bad*
		GSE16446 (Primary ER-negative tumours of anthracycline-treated patients (neoadjuvant))	0 / 0 / 34 / 72 / 1	54 / 107	RFS / DMFS	**0.017**	**8.46 (1.05 – 68.32) Bad**
					OS	0.25	3.46 (0.37 – 32.02)
		GSEE17907 (tumours with amplification of the *ERBB2* locus)	5 / 5 / 24 / 2 / 2	20 / 54	RFS / DMFS	0.79	0.83 (0.21 – 3.34)
		GSE19615	45 / 23 / 17 / 30 / 0	58 / 115	RFS / DMFS	0.98	1.02 (0.29 – 3.52)

		GSE20685	117 / 91 / 69 / 42 / 8	164 / 327	RFS / DMFS	0.12	0.58 (0.29 – 1.16)
					OS	**0.039**	**0.47 (0.23 – 0.98) Good**
			25 / 26 / 20 / 7 / 3	40 / 81	PPS	**0.034**	**0.47 (0.23 – 0.96) Good**
		GSE20711	16 / 32 / 17 / 23 / 0	44 / 90	RFS	0.33	1.56 (0.64 – 3.84)
					OS	0.98	1.02 (0.33 – 3.15)
			4 / 18 / 11 / 9 / 0	20 / 42	PPS	0.26	0.48 (0.13 – 1.76)
		GSE21653	73 / 59 / 21 / 69 / 8	116 / 266	RFS	0.58	0.83 (0.43 – 1.61)
		GSE42568	27 / 28 / 12 / 30 / 7	52 / 104	RFS	**0.02**	**0.41 (0.19 – 0.89) Good**
					OS	**0.001**	**0.22 (0.08 – 0.62) Good**
			6 / 8 / 6 / 10 / 3	16 / 33	PPS	**0.031**	**0.2 (0.04 – 1) Good**
		GSE48390 (breast tumours from Taiwanese women)	27 / 28 / 10 / 8 / 7	40 / 109	RFS	0.97	1.04 (0.15 – 7.39)
					OS	0.99	1.03 (0.06 – 16.41)
		GSE58812 (triple-negative (IHC))	6 / 4 / 13 / 80 / 4	54 / 107	OS	*0.55*	*1.38 (0.48 – 3.97) Bad*
					DMFS	0.96	1.03 (0.36 – 2.93)
		GSE61304	16 / 15 / 11 / 16 / 0	28 / 58	RFS	**0.009**	**0.11 (0.01 – 0.83)**

							**Good**
					DMFS	**0.042**	**0.15 (0.02 – 1.23) Good**
		GSE65194	29 / 17 / 43 / 41 / 0	63 / 130	RFS	0.15	625e7 (0 – Inf)
					DMFS	0.84	1.15 (0.29 – 4.63)
					OS	0.81	1.21 (0.24 – 6.05)
		GSE9195 (ER+ primary breast tumors of patients treated by Tamoxifen in adjuvant setting)	41 / 28 / 7 / 1 / 0	38 / 77	RFS	0.36	0.46 (0.08 – 2.51)
					DMFS	0.89	0.87 (0.12 – 6.19)
	RNA-seq	GSE96058	1504 / 668 / 295 / 309 / 200	1486 / 2976	OS	**0.016**	**0.68 (0.49 – 0.93) Good**
	Protein	Tang_2018	NA	65 *	OS	**0.014**	**2.6 (1.18 – 5.75) Bad**
**HPA**	RNA-seq	TCGA	NA	1075 *	OS	**0.001**	**Bad**
**cBioPortal**	Gene chip	METABRIC	700 / 475 / 224 / 209 / 148 (218 claudin-low / 529 NA / 6 NC)	952 / 2976 (-605 NA)	OS	0.362	
					RFS	*0.073*	*Bad*
	RNA-seq	TCGA PanCancer Atlas	499 / 197 / 78 / 171 / 36 (103 NA)	541 / 1084 (-2 NA)	OS	**0.012**	**Bad**
					PFS	0.227	-
					DSFS	0.273	-
					DFS	0.909	-

*Differentially expressed method is automatic (by median), for all other analysis samples were divided by high (upper quartile) *vs* low (lower quartile) SETD7 expression.

Table S5. Prognosis associated with high *SETD7* in BC subtypes. Significant values are highlighted in bold and strong tendencies (p-val ≤ 0.1) are shown in parenthesis. Log rank test. DFS – Disease free survival; DMFS – Distant metastasis free surviv5al; DSFS – Disease-specific free survival; NA – not available, either because the number of samples were too low for each subgroup or because KM plotter didn't perform the analysis; OS – overall survival; PFS – Progression free survival; PPS – Palliative performance score; RFS – Recurrence/Relapse free survival.

**Online Tool**	**Type of data**	**Datasets**	**Survival**	**Luminal A** p-value (nSETD7 DE / nTotal samples)	**Luminal B** p-value (nSETD7 DE / nTotal samples)	**Her2+** p-value (nSETD7 DE / nTotal samples)	**Basal** p-value (nSETD7 DE / nTotal samples)	**Normal-like** p-value (nSETD7 DE / nTotal samples)	**Claudin-low** p-value (nSETD7 DE / nTotal samples)
KM plotter	Gene chip	All studies	RFS	0.89 (317 / 631)	0.16 (284 / 566)	0.41 (180 / 358)	**0.009 (221 / 442) Bad**	0.64 (18 / 35)	-

			DMFS	**0.027 (130 / 259) Good**	0.93 (92 / 183)	0.64 (104 / 218)	0.27 (142 / 283)	0.73 (8 / 15)	-
			OS	0.41 (112 / 222)	0.47 (100 / 200)	0.091 (100 / 198)	0.33 (148 / 296)	0.99 (14 / 27)	-
			PPS	0.88 (18 / 36)	0.21 (26 / 53)	0.065 (24 / 47) (Good)	0.18 (20 / 38)	NA (NA / 6)	-
		E-MTAB-365	RFS	0.28 (88 / 174)	0.2 (74 / 149)	0.59 (26 / 53)	0.32 (25 / 49)	NA (- / 1)	-
		GSE12276	RFS	0.83 (16 / 34)	0.14 (36 / 72)	0.51 (20 / 38)	0.8 (30 / 59)	NA (- / 1)	-
		GSE16391	RFS	0.14 (14 / 27)	NA (NA / 19)	NA (- / 2)	-	-	-
		GSE16446	RFS / DMFS	-	-	NA (NA / 34)	**0.029 (36 / 72) Bad**	NA (- / 1)	-
			OS	-	-	NA (NA / 34)	0.061 (36 / 72) (Bad)	NA (- / 1)	-

		GSE17907	RFS / DMFS	NA (NA / 5)	NA (NA / 5)	0.77 (12 / 24)	NA (- / 2)	NA (- / 2)	-
		GSE19615	RFS / DMFS	0.53 (22/ 45)	NA (NA / 23)	NA (NA / 17)	0.14 (16 / 30)	-	-
		GSE20685	RFS / DMFS	0.21 (58 / 117)	0.72 (46 / 91)	0.86 (34 / 69)	0.15 (20 / 42)	NA (NA / 8)	-
			OS	0.19 (58 / 177)	0.62 (46 / 91)	0.29 (34 / 69)	0.1 (20 / 42)	NA (NA / 8)	-
			PPS	0.93 (12 / 25)	0.76 (12 / 26)	0.096 (10 / 20)	NA (NA / 7)	NA (NA / 3)	-
		GSE20711	RFS	NA (NA / 16)	0.57 (16 / 32)	NA (NA / 17)	**0.018 (12 / 23) Bad**	-	-
			OS	NA (NA / 16)	0.97 (16 / 32)	NA (NA / 17)	0.095 (12 / 23) (Bad)	-	-
			PPS	NA (NA / 4)	NA (NA / 18)	NA (NA / 11)	NA (NA / 9)	-	-
		GSE21653	RFS	0.66 (36 / 73)	0.075 (30 / 59)	0.7 (10 / 21)	0.46 (34 / 69)	NA (NA / 8)	-

					(Good)				
		GSE42568	RFS	0.47 (14 /27)	0.56 (14 / 28)	NA (NA / 12)	0.2 (16 / 30)	NA (NA / 7)	-
			OS	0.14 (14 /27)	0.1 (14 / 28)	NA (NA / 12)	0.063 (16 / 30) (Good)	NA (NA / 7)	-
			PPS	NA (NA / 6)	NA (NA / 8)	NA (NA / 6)	NA (NA / 10)	NA (NA / 3)	-
		GSE48390	RFS	NA (NA / 27)	NA (NA / 28)	NA (NA / 10)	NA (NA / 8)	NA (NA / 7)	-
			OS	NA (NA / 27)	NA (NA / 28)	NA (NA / 10)	NA (NA / 8)	NA (NA / 7)	-
		GSE61304	RFS	NA (NA / 16)	NA (NA / 15)	NA (NA / 11)	NA (NA / 16)	-	-
			DMFS	NA (NA / 16)	NA (NA / 15)	NA (NA / 11)	NA (NA / 16)	-	-
		GSE65194	RFS	NA (NA / 29)	NA (NA / 17)	NA (NA / 43)	NA (NA 41)	-	-

			DMFS	NA (NA / 29)	NA (NA / 17)	0.68 (22 / 43)	**0.024 (20 / 41) Bad**	-	-
			OS	NA (NA / 29)	NA (NA / 17)	0.9 (22 / 43)	**0.0052 (20 / 41) Bad**	-	-
		GSE9195	RFS	0.15 (20 / 41)	0.06 (14 / 28) (Bad)	NA (NA / 7)	NA (- / 1)	-	-
			DMFS	NA (NA / 41)	**0.045 (14 / 28) Bad**	NA (NA / 7)	NA (- / 1)	-	-
		GSE58812	OS	NA (NA / 6)	NA (NA / 4)	NA (NA / 13)	0.55 (40 / 80)	NA (NA / 4)	-
			DMFS	NA (NA / 6)	NA (NA / 4)	NA (NA / 13)	0.81 (40 / 80)	NA (NA / 4)	-
	RNA-seq	GSE96058	OS	0.31 (751 / 1504)	0.14 (334 / 668)	0.95 (149 / 295)	0.7 (154 / 309)	0.52 (100 / 200)	-
cBioPortal	Gene chip	METABRIC	OS	0.651 (339 / 700)	0.514 (232 / 475)	0.513 (110 / 224)	0.879 (99 / 209)	0.435 (70 / 148)	0.511 (99 / 218)

			RFS	0.721 (339 / 700)	0.111 (231 / 475)	0.967 (110 / 224)	0.830 (99 / 209)	0.392 (70 / 148)	0.133 (99 / 218)
	RNA-seq	TCGA PanCancer Atlas	OS	**0.044 (249 / 499) Bad**	0.712 (99 / 197)	0.0959 (39 / 78) (Bad)	0.456 (85 / 171)	0.381 (18 / 36)	-
			PFS	**0.0319 (249 / 499) Bad**	0.521 (99 / 197)	0.0511 (39 / 78) (Bad)	0.125 (85 / 171)	0.696 (18 / 36)	-
			DFS	0.701 (249 / 499)	0.577 (99 / 197)	0.154 (39 / 78)	0.0626 (85 / 171) (Bad)	0.371 (18 / 36)	-
			DSFS	0.0785 (249 / 499) (Bad)	0.370 (99 / 197)	0.169 (39 / 78)	0.610 (85 / 171)	0.967 (18 / 36) (Bad)	-

Table S6. Prognosis associated with high *SETD7* in BC considering therapy. Significant values are highlighted in bold (Log rank test p-val ≤ 0.05). CI – Confidence Interval, DFS – Disease Free Survival; DMFS – Distant metastasis free survival; DSFS – Disease-specific Free Survival; HR – Hazard Ratio, NA – Not Available, either because the number of samples were too low for each subgroup or because KM plotter didn't perform the analysis; OS – Overall Survival; PFS – Progression Free Survival; PPS – Palliative Performance Score; RFS – Recurrence/Relapse Free Survival.

**Online Tool**	**Type of data**	**Datasets**	**Survival**	**No therapy** p-value (nSETD7 DE / nTotal samples) HR (CI) / prognosis	**Hormone plus Chemotherapy** p-value (nSETD7 DE / n Total samples) HR (CI) / prognosis	**Hormone therapy** p-value (nSETD7 DE / n Total samples) HR (CI) / prognosis	**Chemotherapy** p-value (nSETD7 DE / n Total samples) HR (CI) / prognosis
KM plotter	Gene chip	All studies reporting treatment	RFS	0.18 (32 / 62)<br>0.39 (0.09 – 1.64)	0.052 (76 / 153)<br>0.33 (0.1 – 1.07)	0.99 (90 / 181)<br>0.99 (0.39 – 2.5)	**0.0006 (106 / 211)**<br>**4.26 (1.73 – 10.5)**<br>**Bad**
			DMFS	0.81 (4 / 7)<br>1.41 (0.08 – 23.57)	0.28 (28 / 55)<br>3.24 (0.34 – 31.19)	0.3 (51 / 99)<br>0.32 (0.03 – 3.06)	**0.0012 (84 / 168)**<br>**5.98 (1.75 – 20.47)**<br>**Bad**

			OS	-	-	-	0.25 (54 / 107)<br>3.46 (0.37 – 32.02)
		GSE16391	RFS	-	NA (NA / 18)<br>-	0.14 (16 / 30)<br>4.5 (0.5 – 40.38)	**-**
		GSE16446	RFS / DMFS	-	-	-	**0.017 (54 / 107)**<br>**8.46 (1.05 -68.32)**<br>**Bad**
			OS				0.25 (54 / 107)<br>3.46 (0.37 – 32.02)
		GSE17907	RFS / DMFS	-	NA (NA / 13)	NA (NA / 1)	0.6 (12 / 23)<br>0.65 (0.13 – 3.25)
		GSE19615	RFS / DMFS	NA (NA / 7)	0.15 (20 / 42)<br>171e7 (0 – Inf)	NA (NA / 21)	0.15 (20 / 38)<br>4.41 (0.49 – 39.51)
		GSE21653	RFS	0.16 (28 / 55)<br>0.31 (0.06 – 1.73)	0.13 (40 / 80)<br>0.41 (0.12 -1.35)	0.89 (26 / 52)<br>1.12 (0.22 – 5.61)	0.13 (22 / 43)<br>2.8 (0.69 – 11.29)
		GSE9195	RFS	-	-	0.36 (38 / 77)<br>0.46 (0.08 – 2.51)	-
			DMFS	-	-	0.89 (38 / 77)	-

						0.87 (0.12 – 6.19)	
		GSE42568	RFS	-	-	-	**0.02 (52 / 104)** **0.41 (0.19 – 0.89)** **Bad**
			OS	-	-	-	**0.0016 (52 / 104)** **0.22 (0.08 – 0.62)** **Bad**
			PPS	-	-	-	0.28 (8 / 17)
	RNA-seq	GSE96058	OS	**0.016 (156 / 313)** **0.36 (0.15 – 0.86)** **Good**	0.088 (429 / 854) 0.44 (0.17 – 1.16)	0.68 (724 / 1448) 0.92 (0.6 – 1.39)	0.8 (170 / 340) 0.91 (0.41 – 1.98)
cBioPortal	Gene Chip	METABRIC	RFS	0.381 (258 / 543)	0.283 (91 / 191)	**0.0325 (495/1025)** **Bad**	0.965 (107 / 221)
			OS	0.458 (259 / 543)	0.625 (91 / 191)	0.325 (495 / 1025)	0.933 (107 / 221)
	RNA-seq	TCGA PanCancer Atlas	OS	**0.0295 (537 / 1077)** **Bad**	Any: 6	-	-
			PFS	0.279 (536 / 1077)	Any: NA (NA / 6)	-	-

			DSFS	0.345 (533 / 1077)	Any: NA (NA / 6)	-	-
			DFS	0.891 (454 / 1077)	Any: NA (NA / 6)	-	-

Table S7. Prognosis associated with high *SETD7* in BC considering stage. Significant values are highlighted in bold (Log rank test p-val ≤ 0.05) and strong tendencies (Log rank test p-val ≤ 0.1) are shown in italic. DFS – Disease Free Survival; DSFS – Disease-specific Free Survival; NA – Not Available, because the number of samples were too low for each subgroup; OS – Overall Survival; PFS – Progression Free Survival; RFS – Recurrence/Relapse Free Survival.

**Online tool**	**Type of data**	**Dataset**	**Survival**	**Stage 0** p-value (nSETD7 DE / nTotal samples)	**Stage I** p-value (nSETD7 DE / nTotal samples)	**Stage II** p-value (nSETD7 DE / nTotal samples)	**Stage III** p-value (nSETD7 DE / nTotal samples)	**Stage IV** p-value (nSETD7 DE / nTotal samples)
HPA	RNA-seq	TCGA	OS	-	*0.095 (180 / 180) Bad*	**0.00027 (609 / 609) Bad**	0.84 (243 / 243)	0.90 (20 / 20)
cBioPortal	Gene chip	METABRIC	OS	NA (NA / 24)	0.634 (237 / 630)	0.668 (400 / 979)	*0.0957 (57 / 144) Good*	NA (NA / 11)

			RFS	NA (NA / 24)	0.249 (236 / 630)	**0.0396 (400 / 979) Bad**	0.217 (57 / 144)	NA (NA / 11)
	RNA-seq	TCGA PanCancer Atlas	OS	-	0.332 (138 / 277)	**0.0283 (313 / 628) Bad**	0.520 (69 / 137)	0.633 (19 / 39)
			DFS	-	0.567 (123 / 277)	**0.0569 (312 / 628) Bad**	0.624 (57 / 137)	0.241 (9 / 39)
			PFS	-	0.612 (137 / 277)	0.120 (313 / 628)	0.991 (69 / 137	0.728 (19 / 39)
			DSFS	-	0.877 (137 / 277)	0.325 (270 / 628)	0.685 (67 / 137)	0.879 (19 / 39)
KM plotter	Protein	Tang_2018	OS	-	NA (6 / 6)	**0.036 (46 / 46) Bad**	NA (13 / 13)	-

Table S8. Prognosis associated with high *SETD7* in BC considering grade. Significant values are highlighted in bold (Log rank test p-val ≤ 0.05) and strong tendencies (Log rank test p-val ≤ 0.1) are shown in italic. CI – Confidence Interval, DMFS – Distant Metastasis Free Survival; HR – Hazard Ratio, NA – Not Available, either because the number of samples were too low for each subgroup or simply because KM plotter didn't perform the analysis; OS – Overall Survival; PPS – Palliative Performance Score; RFS – Recurrence/Relapse Free Survival.

**Online Tool**	**Type of data**	**Datasets**	**Survival**	**Grade I**<br>p-value (nSETD7 DE / nTotal samples)<br>HR (CI) / prognosis	**Grade II**<br>p-value (nSETD7 DE / nTotal samples)<br>HR (CI) / prognosis	**Grade III**<br>p-value (nSETD7 DE / nTotal samples)<br>HR (CI) / prognosis
KM plotter	Gene chip	All studies reporting Grade status	RFS	**0.03 (56 / 113)**<br>**0 (0 – Inf) Good**	0.8 (121 / 243)<br>1.09 (0.56 – 2.14)	0.74 (242 / 481)<br>0.93 (0.62 – 1.41)
			DMFS	NA (NA / 44)<br>-	0.5 (46 / 91)<br>1.54 (0.43 – 5.44)	0.093 (116 / 234)<br>1.98 (0.88 – 4.44)
			OS	0.48 (12 / 26)<br>2.35 (0.2 – 27.18)	0.82 (32 / 64)<br>0.82 (0.14 – 4.9)	*0.061 (102 / 204)*<br>*0.52 (0.26 – 1.04) Good*
			PPS	0.69 (4 / 6)<br>1.62 (0.14 – 18.31)	0.69 (6 / 13)<br>0.62 (0.05 – 7)	**0.0022 (36 / 72)**<br>**0.24 (0.09 – 0.64) Good**
		GSE16391	RFS	NA (NA / 2)	0.14 (16 / 30)	NA (NA / 16)

				-	-	-
		GSE16446	RFS / DMFS	NA (NA / 2)<br>-	NA (NA / 19)<br>-	**0.013 (40 / 81)**<br>**173e7 (0 - Inf) Bad**
			OS	NA (NA / 2)<br>-	NA (NA / 19)<br>-	0.13 (40 / 81)<br>139e7 (0 – Inf)
		GSEE17907	RFS / DMFS	-	NA (NA /8)<br>-	0.49 (14 / 29)<br>-
		GSE19615	RFS / DMFS	NA (NA / 23)<br>-	*0.06 (14 / 28)*<br>*195e7 (0 – Inf) Bad*	0.63 (32 / 64)<br>1.54 (0.26 – 9.22)
		GSE20711	RFS	NA (NA / 13)<br>-	NA (NA / 5)<br>-	0.37 (36 / 70)<br>1.55 (0.59 – 4.11)
			OS	NA (NA / 13)<br>-	NA (NA / 5)<br>-	0.63 (36 / 70)<br>1.34 (0.41 – 4.4)
			PPS	NA (NA / 3)<br>-	NA (NA / 2)<br>-	0.21 (18 / 37)<br>0.4 (0.09 – 1.75)
		GSE21653	RFS	*0.082 (22 / 43)*<br>*0 (0 – Inf) Good*	0.79 (38 / 77)<br>1.16 (0.39 – 3.47)	0.92 (54 / 107)<br>0.95 (0.4 – 2.3)

		GSE42568	RFS	NA (NA/ 11)<br>-	0.79 (20 / 40)<br>0.83 (0.2 – 3.36)	0.2 (26 / 53)<br>0.51 (0.17 – 1.47)
			OS	NA (NA/ 11)<br>-	0.57 (20 / 40)<br>0.6 (0.1 – 3.6)	**0.02 (26 / 53)**<br>**0.22 (0.06 – 0.89) Good**
			PPS	NA (NA / 3)<br>-	NA (NA / 8)<br>-	**0.04 (12 / 22)**<br>**0.19 (0.03 – 1.09) Good**
		GSE61304	RFS	NA (NA / 5)<br>-	NA (NA / 16)<br>-	**0.042 (18 / 37)**<br>**0.09 (0.01 – 0.74) Good**
			DMFS	NA (NA / 5)<br>-	NA (NA / 16)<br>-	0.14 (19 / 36)<br>0.23 (0.03 – 1.94)
		GSE9195	RFS	NA (NA / 14)<br>-	NA (NA / 20)<br>-	0.84 (12 / 24)<br>0.82 (0.11 – 5.83)
			DMFS	NA (NA / 14)<br>-	NA (NA / 20)<br>-	0.6 (12 / 24)<br>1.88 (0.17 – 20.82)
	RNA-seq	GSE96058	OS	0.35 (224 / 449)<br>0.52 (0.13 – 2.08)	0.18 (698 / 1394)<br>0.7 (0.41 – 1.18)	0.9 (536 / 1074)<br>0.97 (0.65 – 1.46)
	Protein	Tang_2018	OS	NA (8 / 8)<br>-	NA (19 / 19)<br>-	**0.016 (28 / 28)**<br>**3.39 (1.14 – 10.14) Bad**

cBioPortal	Gene chip	METABRIC	OS	0.516 (83 / 214)	0.745 (370 / 976)	0.421 (463 / 1198)
			RFS	0.863 (83 / 214)	0.186 (370 / 976)	0.309 (462 / 1198)

Table S9. Prognosis associated with high *SETD7* in BC considering lymph node status. Significant values are highlighted in bold (Log rank test p-val ≤ 0.05) and strong tendencies (Log rank test p-val ≤ 0.1) are shown in italic. CI – Confidence Interval, DFS – Disease Free Survival; DFSF – Disease-specific Free Survival; HR – Hazard Ratio, NA – Not Available, either because the number of samples were too low for each subgroup or because the analysis wasn't processed by KM plotter; OS – Overall Survival; PFS – Progression Free Survival; RFS – Recurrence/Relapse Free Survival.

**Online Tool**	**Type of data**	**Dataset**	**Survival**	**Lymph node status**	
				**Positive** p-value (nSETD7 DE / nTotal samples) HR (CI) / Prognosis	**Negative** p-value (nSETD7 DE / nTotal samples) HR (CI) / Prognosis

KM plotter	Gene chip data (EGA and GEO)	All studies reporting lymph node status	RFS	0.11 (408 / 814)<br>0.76 (0.54 – 1.07)	0.74 (288 / 574)<br>0.91 (0.52 – 1.58)
			DMFS	0.19 (130 / 261)<br>1.58 (0.79 – 3.18)	0.39 (120 / 240)<br>0.62 (0.2 – 1.89)
			OS	*0.059 (116 / 230)*<br>*0,53 (0.28 – 1.03) Good*	0.91 (90 / 180)<br>0.93 (0.28 – 3.06)
			PPS	**0.00013 (38 / 76)**<br>**0.15 (0.05 – 0.45)**<br>**Good**	0.45 (12 / 23)<br>0.5 (0.08 – 3.05)
		E-MTAB-365	RFS	0.96 (142 / 285)<br>1.02 (0.54 – 1.9)	0.43 (68 / 134)<br>0.71 (0.31 – 1.65)
		GSE16391	RFS	0.14 (16 / 30)<br>4.53 (0.5 – 40.94)	NA (NA / 18)<br>-
		GSE16446	RFS / DMFS	*0.053 (30 / 59)*<br>*6.39 (0.74 – 54.99) Bad*	0.5 (24 / 48)<br>2.18 (0.21 – 22.2)
			OS	0.48 (30 / 59)<br>2.32 (0.21 – 25.8)	0.31 (24 / 48)<br>114e7 (0 – Inf)
		GSEE17907	RFS / DMFS	0.51 (12 / 24)	NA (NA / 11)

				0.55 (0.09 – 3.33)	-
		GSE19615	RFS / DMFS	0.37 (26 / 53)<br>2.15 (0.39 – 11.74)	0.31 (32 / 62)<br>0.33 (0.03 – 3.15)
		GSE20711	RFS	0.99 (30 / 59)<br>1 (0.36 – 2.75)	0.11 (14 / 29)<br>4.99 (0.56 – 44.83)
			OS	0.45 (30 / 59)<br>0.61 (0.17 – 2.18)	0.14 (14 / 29)<br>175e7 (0 – Inf)
			PPS	*0.055 (18 / 35)*<br>*0.26 (0.06 – 1.12) Good*	NA (NA / 7)<br>-
		GSE21653	RFS	0.77 (60 / 119)<br>0.88 (0.39 – 2.01)	0.71 (54 / 108)<br>0.79 (0.23 – 2.74)
		GSE42568	RFS	0.4 (30 / 59)<br>0.69 (0.29 – 1.64)	**0.042 (22 / 45)**<br>**0.14 (0.01 – 1.26) Good**
			OS	**0.04 (30 / 59)**<br>**0.33 (0.11 – 1) Good**	**0.026 (22 / 45)**<br>**0 (0 – Inf) Good**
			PPS	**0.029 (14 / 28)**<br>**0.18 (0.03 – 0.98)**<br>**Good**	NA (NA / 5)<br>-

		GSE61304	RFS	*0.096 (18 / 37)*<br>*0.28 (0.06 – 1.39) Good*	0.19 (11 / 20)<br>0 (0 – Inf)
			DMFS	0.3 (18 / 37)<br>0.42 (0.08 – 2.27)	0.19 (11 / 20)<br>0 (0 – Inf)
		GSE65194	RFS	NA (NA / 53)	NA (NA / 58)
			OS	0.31 (26 / 53)<br>3.08 (0.32 – 29.71)	NA (NA / 58)<br>-
			DMFS	0.51 (26 / 53)<br>1.65 (0.37 – 7.42)	0.16 (28 / 58)<br>0 (0 – Inf)
		GSE9195	RFS	0.28 (18 / 36)<br>0.41 (0.07 – 2.23)	NA (NA / 41)<br>-
			DMFS	0.81 (18 / 36)<br>0.79 (0.11 – 5.67)	NA (NA / 41)<br>-
	RNA-seq	GSE96058	OS	0.47 (911 / 1067)<br>0.85 (0.55 – 1.32)	0.055 (534 / 1820)<br>0.63 (0.39 – 1.01)
	Protein	Tang_2018	OS	0.8 (27 / 27)<br>0.85 (0.24 – 3.01)	**0.00019 (37 / 37)**<br>**7.28 (2.17 – 24.47) Bad**

cBioPortal	Gene Chip	METABRIC	RFS	**0.038 (455 / 1047) Bad**	0.680 (496 / 1196)
			OS	0.423 (455 / 1047)	0.766 (497 / 1196)
	RNA-seq	TCGA PanCancer Atlas	OS	0.116 (343 / 687)	0.806 (17 / 33)
			DFS	0.337 (299 / 687)	0.407 (11 / 33)
			DSFS	0.447 (340 / 687)	0.806 (17 / 33)
			PFS	0.772 (342 / 687)	0.468 (17 / 33)

References

1. Dhayalan, A.; Kudithipudi, S.; Rathert, P.; Jeltsch, A. Specificity Analysis-Based Identification of New Methylation Targets of the SET7/9 Protein Lysine Methyltransferase. *Chem. Biol.* **2011**, *18* (1), 111–120.
2. Ko, S.; Ahn, J.; Song, C. S.; Kim, S.; Knapczyk-Stwora, K.; Chatterjee, B. Lysine Methylation and Functional Modulation of Androgen Receptor by Set9 Methyltransferase. *Mol. Endocrinol.* **2011**, *25* (3), 433–444.
3. Gaughan, L.; Stockley, J.; Wang, N.; McCracken, S. R. C.; Treumann, A.; Armstrong, K.; Shaheen, F.; Watt, K.; McEwan, I. J.; Wang, C.; et al. Regulation of the Androgen Receptor by SET9-Mediated Methylation. *Nucleic Acids Res.* **2011**, *39* (4), 1266–1279.
4. Shen, C.; Wang, D.; Liu, X.; Gu, B.; Du, Y.; Wei, F. Z.; Cao, L. L.; Song, B.; Lu, X.; Yang, Q.; et al. SET7/9 Regulates Cancer Cell Proliferation by Influencing β-Catenin Stability. *FASEB J.* **2015**, *29* (10), 4313–4323.
5. Estève, P. O.; Chin, H. G.; Benner, J.; Feehery, G. R.; Samaranayake, M.; Horwitz, G. A.; Jacobsen, S. E.; Pradhan, S.

Regulation of DNMT1 Stability through SET7-Mediated Lysine Methylation in Mammalian Cells. *Proc. Natl. Acad. Sci. U. S. A.* **2009**, *106* (13), 5076–5081.

6. Subramanian, K.; Jia, D.; Kapoor-Vazirani, P.; Powell, D. R.; Collins, R. E.; Sharma, D.; Peng, J.; Cheng, X.; Vertino, P. M. Regulation of Estrogen Receptor Alpha by the SET7 Lysine Methyltransferase. *Mol. Cell* **2008**, *30* (3), 336–347.
7. Xie, Q.; Bai, Y.; Wu, J.; Sun, Y.; Wang, Y.; Zhang, Y.; Mei, P.; Yuan, Z. Methylation-Mediated Regulation of E2F1 in DNA Damage-Induced Cell Death. *J. Recept. Signal Transduct.* **2011**, *31* (2), 139–146.
8. Kontaki, H.; Talianidis, I. Lysine Methylation Regulates E2F1-Induced Cell Death. *Mol. Cell* **2010**, *39* (1), 152–160.
9. Calnan, D. R.; Webb, A. E.; White, J. L.; Stowe, T. R.; Goswami, T.; Shi, X.; Espejo, A.; Bedford, M. T.; Gozani, O.; Gygi, S. P.; et al. Methylation by Set9 Modulates FoxO3 Stability and Transcriptional Activity. *Aging (Albany. NY).* **2012**, *4* (7), 462–479.
10. Xie, Q.; Hao, Y.; Tao, L.; Peng, S.; Rao, C.; Chen, H.; You, H.; Dong, M. Q.; Yuan, Z. Lysine Methylation of FOXO3 Regulates Oxidative Stress-Induced Neuronal Cell Death. *EMBO Rep.* **2012**, *13* (4), 371.
11. Fu, L.; Wu, H.; Cheng, S. Y.; Gao, D.; Zhang, L.; Zhao, Y. Set7 Mediated Gli3 Methylation Plays a Positive Role in the Activation of Sonic Hedgehog Pathway in Mammals. *Elife* **2016**, *5* (MAY2016), e15690.
12. Kim, Y.; Nam, H. J.; Lee, J.; Park, D. Y.; Kim, C.; Yu, Y. S.; Kim, D.; Park, S. W.; Bhin, J.; Hwang, D.; et al. Methylation-Dependent Regulation of HIF-1α Stability Restricts Retinal and Tumour Angiogenesis. *Nat. Commun.* **2016**, *7*, 10347.
13. Pagans, S.; Kauder, S. E.; Kaehlcke, K.; Sakane, N.; Schroeder, S.; Dormeyer, W.; Trievel, R. C.; Verdin, E.; Schnolzer, M.; Ott, M. Set7/9 (KMT7) Binds HIV-1 TAR RNA, Monomethylates Tat and Enhances Tat-Dependent HIV Transcription. *Cell Host Microbe* **2010**, *7* (3), 234.

14. Ali, I.; Ramage, H.; Boehm, D.; Dirk, L. M. A.; Sakane, N.; Hanada, K.; Pagans, S.; Kaehlcke, K.; Aull, K.; Weinberger, L.; et al. The HIV-1 Tat Protein Is Monomethylated at Lysine 71 by the Lysine Methyltransferase KMT7. *J. Biol. Chem.* **2016**, *291* (31), 16240.
15. Masatsugu, T.; Yamamoto, K. Multiple Lysine Methylation of PCAF by Set9 Methyltransferase. *Biochem. Biophys. Res. Commun.* **2009**, *381* (1), 22–26.
16. Vasileva, E.; Shuvalov, O.; Petukhov, A.; Fedorova, O.; Daks, A.; Nader, R.; Barlev, N. KMT Set7/9 Is a New Regulator of Sam68 STAR-Protein. *Biochem. Biophys. Res. Commun.* **2020**, *525* (4), 1018–1024.
17. Kim, S. K.; Lee, H.; Han, K.; Kim, S. C.; Choi, Y.; Park, S. W.; Bak, G.; Lee, Y.; Choi, J. K.; Kim, T. K.; et al. SET7/9 Methylation of the Pluripotency Factor LIN28A Is a Nucleolar Localization Mechanism That Blocks Let-7 Biogenesis in Human ESCs. *Cell Stem Cell* **2014**, *15* (6), 735.
18. Balasubramaniyan, N.; Ananthanarayanan, M.; Suchy, F. J. Direct Methylation of FXR by Set7/9, a Lysine Methyltransferase, Regulates the Expression of FXR Target Genes. *Am. J. Physiol. - Gastrointest. Liver Physiol.* **2012**, *302* (9), G937.
19. Kassner, I.; Andersson, A.; Fey, M.; Tomas, M.; Ferrando-May, E.; Hottiger, M. O. SET7/9-Dependent Methylation of ARTD1 at K508 Stimulates Poly-ADP-Ribose Formation after Oxidative Stress. *Open Biol.* **2013**, *3* (10), 120173.
20. Maganti, A. V.; Maier, B.; Tersey, S. A.; Sampley, M. L.; Mosley, A. L.; Özcan, S.; Pachaiyappan, B.; Woster, P. M.; Hunter, C. S.; Stein, R.; et al. Transcriptional Activity of the Islet β Cell Factor Pdx1 Is Augmented by Lysine Methylation Catalyzed by the Methyltransferase Set7/9. *J. Biol. Chem.* **2015**, *290* (15), 9812.
21. Aguilo, F.; Li, S. De; Balasubramaniyan, N.; Sancho, A.; Benko, S.; Zhang, F.; Vashisht, A.; Rengasamy, M.; Andino, B.; Chen, C. hung; et al. Deposition of 5-Methylcytosine on Enhancer RNAs Enables the Coactivator Function of PGC-1α. *Cell Rep.*

2016, *14* (3), 479–492.

22. Cho, H. S.; Suzuki, T.; Dohmae, N.; Hayami, S.; Unoki, M.; Yoshimatsu, M.; Toyokawa, G.; Takawa, M.; Chen, T.; Kurash, J. K.; et al. Demethylation of RB Regulator MYPT1 by Histone Demethylase LSD1 Promotes Cell Cycle Progression in Cancer Cells. *Cancer Res.* **2011**, *71* (3), 655–660.
23. Carr, S. M.; Munro, S.; Kessler, B.; Oppermann, U.; La Thangue, N. B. Interplay between Lysine Methylation and Cdk Phosphorylation in Growth Control by the Retinoblastoma Protein. *EMBO J.* **2011**, *30* (2), 317–327.
24. Munro, S.; Khaire, N.; Inche, A.; Carr, S.; La Thangue, N. B. Lysine Methylation Regulates the PRb Tumour Suppressor Protein. *Oncogene* **2010**, *29* (16), 2357–2367.
25. Ea, C. K.; Baltimore, D. Regulation of NF-KB Activity through Lysine Monomethylation of P65. *Proc. Natl. Acad. Sci. U. S. A.* **2009**, *106* (45).
26. Yang, X. D.; Huang, B.; Li, M.; Lamb, A.; Kelleher, N. L.; Chen, L. F. Negative Regulation of NF-KappaB Action by Set9-Mediated Lysine Methylation of the RelA Subunit. *EMBO J.* **2009**, *28* (8), 1055–1066.
27. Hong, X.; Huang, H.; Qiu, X.; Ding, Z.; Feng, X.; Zhu, Y.; Zhuo, H.; Hou, J.; Zhao, J.; Cai, W.; et al. Targeting Posttranslational Modifications of RIOK1 Inhibits the Progression of Colorectal and Gastric Cancers. *Elife* **2018**, *7*, e29511.
28. Song, H.; Chu, J. W.; Park, S. C.; Im, H.; Park, I. G.; Kim, H.; Lee, J. M. Isoform-specific Lysine Methylation of RORα2 by SETD7 Is Required for Association of the TIP60 Coactivator Complex in Prostate Cancer Progression. *Int. J. Mol. Sci.* **2020**, *21* (5), 1622.
29. Hamidi, T.; Singh, A. K.; Veland, N.; Vemulapalli, V.; Chen, J.; Hardikar, S.; Bao, J.; Fry, C. J.; Yang, V.; Lee, K. A.; et al. Identification of Rpl29 as a Major Substrate of the Lysine Methyltransferase Set7/9. *J. Biol. Chem.* **2018**, *293* (33), 12770–12780.

30. Liu, X.; Wang, D.; Zhao, Y.; Tu, B.; Zheng, Z.; Wang, L.; Wang, H.; Gu, W.; Roeder, R. G.; Zhu, W. G. Methyltransferase Set7/9 Regulates P53 Activity by Interacting with Sirtuin 1 (SIRT1). *Proc. Natl. Acad. Sci. U. S. A.* **2011**, *108* (5), 1925–1930.
31. Elkouris, M.; Kontaki, H.; Stavropoulos, A.; Antonoglou, A.; Nikolaou, K. C.; Samiotaki, M.; Szantai, E.; Saviolaki, D.; Brown, P. J.; Sideras, P.; et al. SET9-Mediated Regulation of TGF-β Signaling Links Protein Methylation to Pulmonary Fibrosis. *Cell Rep.* **2016**, *15* (12), 2733–2744.
32. Fang, L.; Zhang, L.; Wei, W.; Jin, X.; Wang, P.; Tong, Y.; Li, J.; Du, J. X.; Wong, J. A Methylation-Phosphorylation Switch Determines Sox2 Stability and Function in ESC Maintenance or Differentiation. *Mol. Cell* **2014**, *55* (4), 537–551.
33. Stark, G. R.; Wang, Y.; Lu, T. Lysine Methylation of Promoter-Bound Transcription Factors and Relevance to Cancer. *Cell Res.* **2011**, *21* (3), 375.
34. Wang, D.; Zhou, J.; Liu, X.; Lu, D.; Shen, C.; Du, Y.; Wei, F. Z.; Song, B.; Lu, X.; Yu, Y.; et al. Methylation of SUV39H1 by SET7/9 Results in Heterochromatin Relaxation and Genome Instability. *Proc. Natl. Acad. Sci. U. S. A.* **2013**, *110* (14), 5516–5521.
35. Couture, J. F.; Collazo, E.; Hauk, G.; Trievel, R. C. Structural Basis for the Methylation Site Specificity of SET7/9. *Nat. Struct. Mol. Biol. 2006 132* **2006**, *13* (2), 140–146.
36. Kouskouti, A.; Scheer, E.; Staub, A.; Tora, L.; Talianidis, I. Gene-Specific Modulation of TAF10 Function by SET9-Mediated Methylation. *Mol. Cell* **2004**, *14* (2), 175–182.
37. Ivanov, G. S.; Ivanova, T.; Kurash, J.; Ivanov, A.; Chuikov, S.; Gizatullin, F.; Herrera-Medina, E. M.; Rauscher, F.; Reinberg, D.; Barlev, N. A. Methylation-Acetylation Interplay Activates P53 in Response to DNA Damage. *Mol. Cell. Biol.* **2007**, *27* (19), 6756–6769.

38. Oudhoff, M. J.; Freeman, S. A.; Couzens, A. L.; Antignano, F.; Kuznetsova, E.; Min, P. H.; Northrop, J. P.; Lehnertz, B.; Barsyte-Lovejoy, D.; Vedadi, M.; et al. Control of the Hippo Pathway by Set7-Dependent Methylation of Yap. *Dev. Cell* **2013**, *26* (2), 188–194.

39. Zhang, W. J.; Wu, X. N.; Shi, T. T.; Xu, H. T.; Yi, J.; Shen, H. F.; Huang, M. F.; Shu, X. Y.; Wang, F. F.; Peng, B. L.; et al. Regulation of Transcription Factor Yin Yang 1 by SET7/9-Mediated Lysine Methylation. *Sci. Rep.* **2016**, *6*.

40. Wu, X. N.; Shi, T. T.; He, Y. H.; Wang, F. F.; Sang, R.; Ding, J. C.; Zhang, W. J.; Shu, X. Y.; Shen, H. F.; Yi, J.; et al. Methylation of Transcription Factor YY2 Regulates Its Transcriptional Activity and Cell Proliferation. *Cell Discov.* **2017**, *3*, 17035.
