# Supplementary material for: SETD7 Expression Is Associated with Breast Cancer Survival Outcomes for Specific Molecular Subtypes: A Systematic Analysis of Publicly Available Datasets
Source: Cancers (Basel). 2022 Dec 7;14(24):6029. doi: 10.3390/cancers14246029 (PMC9775934; doi:10.3390/cancers14246029)
Supplement: Supplementary file 1 [file cancers-14-06029-s001.zip › SupplementaryFigures.pdf]

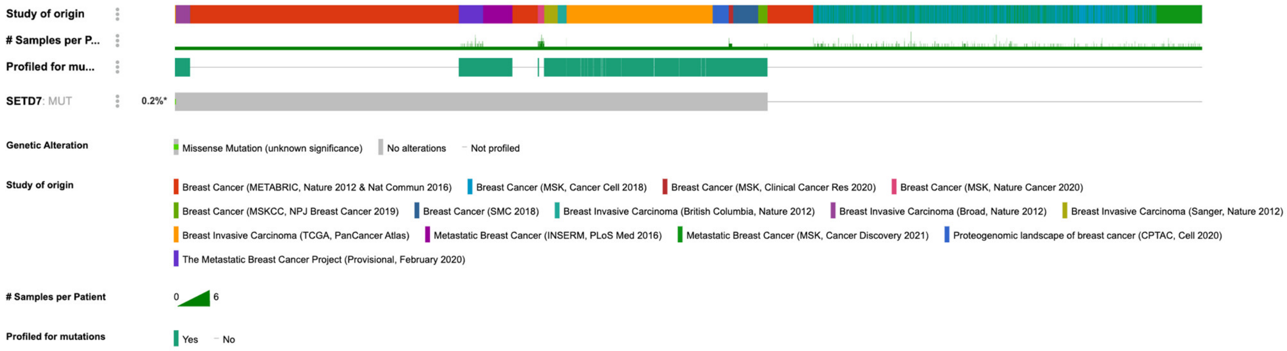

(**a**)

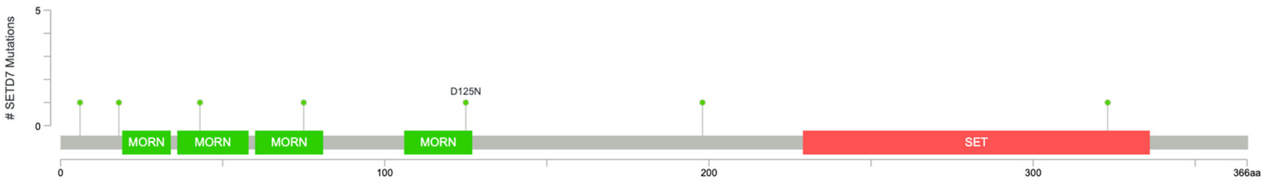

(**b**)

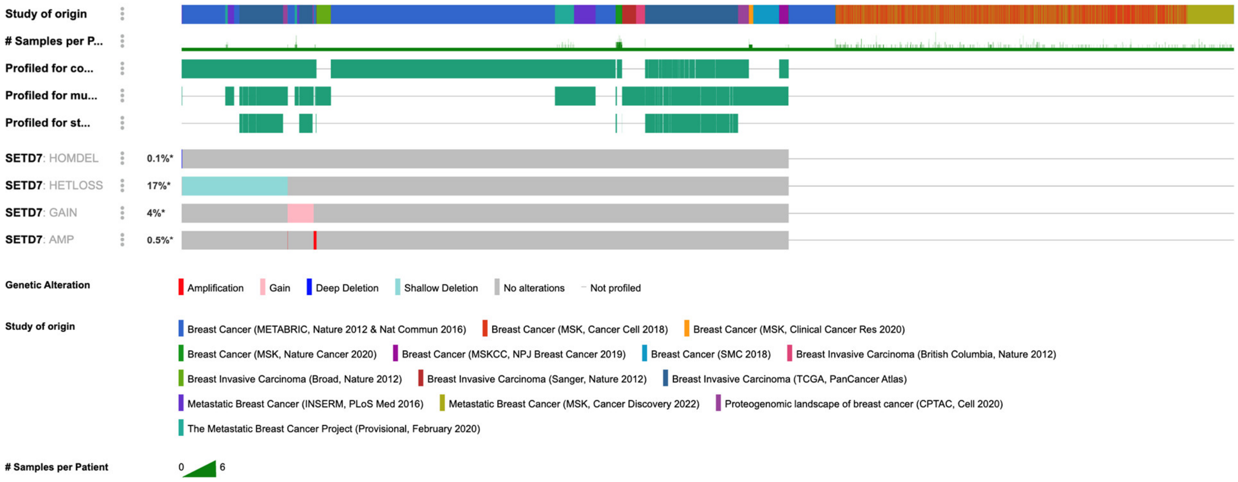

(**c**)

Figure S1. *SETD7* genomic alterations in breast cancer using cBioPortal. (**a**) Oncogrid for mutations; (**b**) *SETD7* mutation sites (lolipop) (**c**)**.** Oncogrid for copy number alterations.

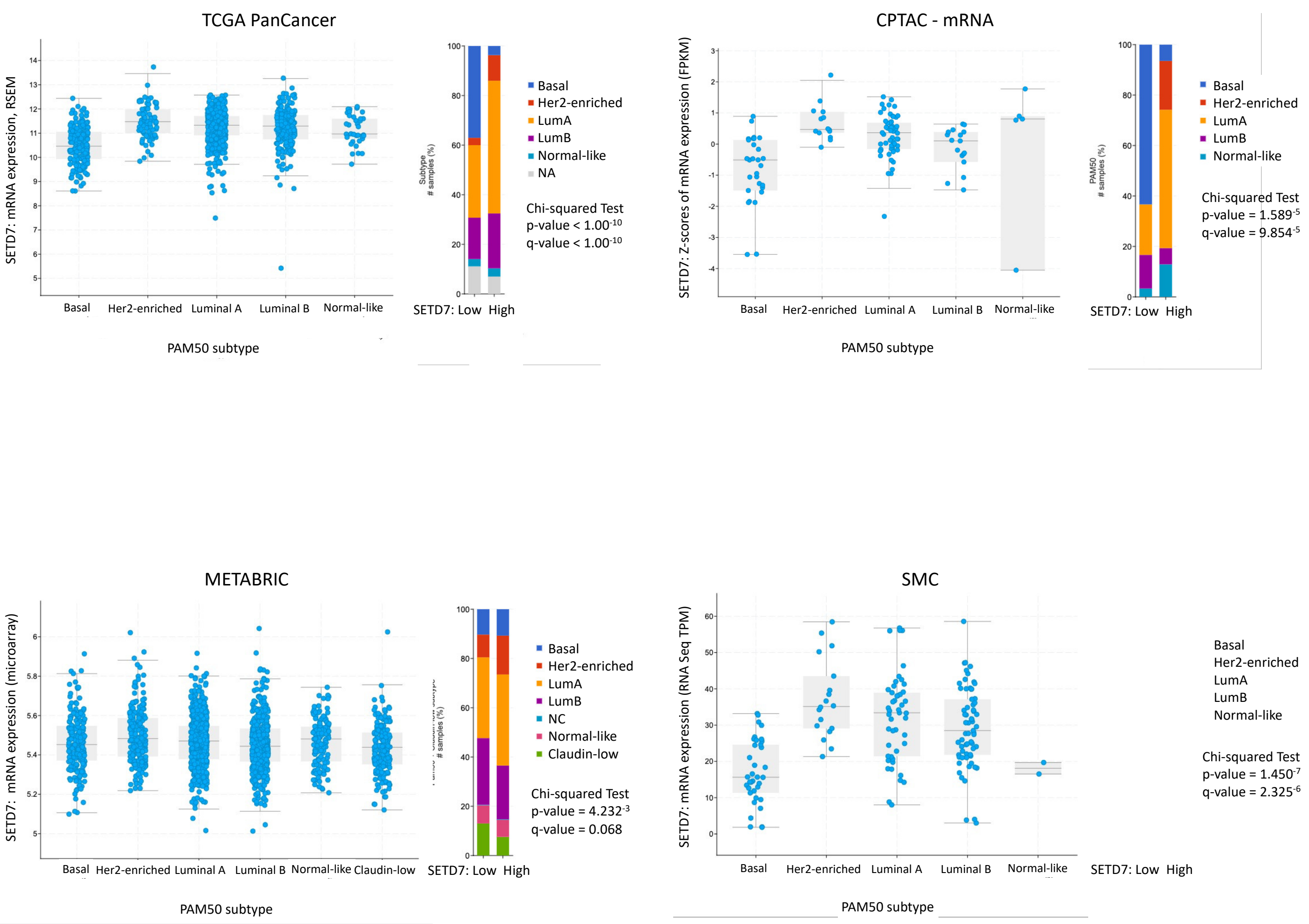

Figure S2. *SETD7* mRNA expression in breast cancer PAM50 subtypes using cBioPortal datasets.

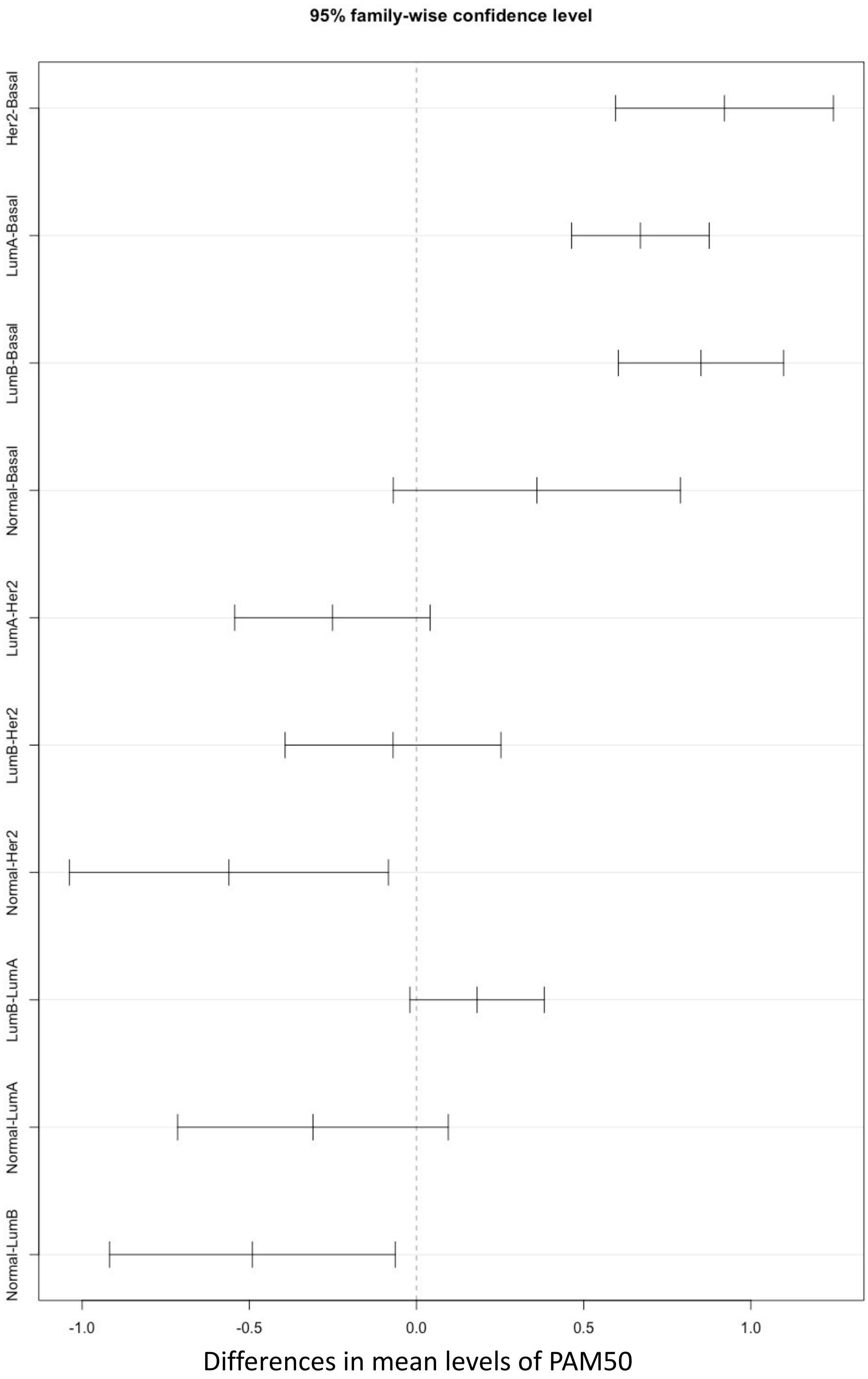

Figure S3. Mean differences of each comparison done in Figure 1B and the 95% confidence intervals associated with them.

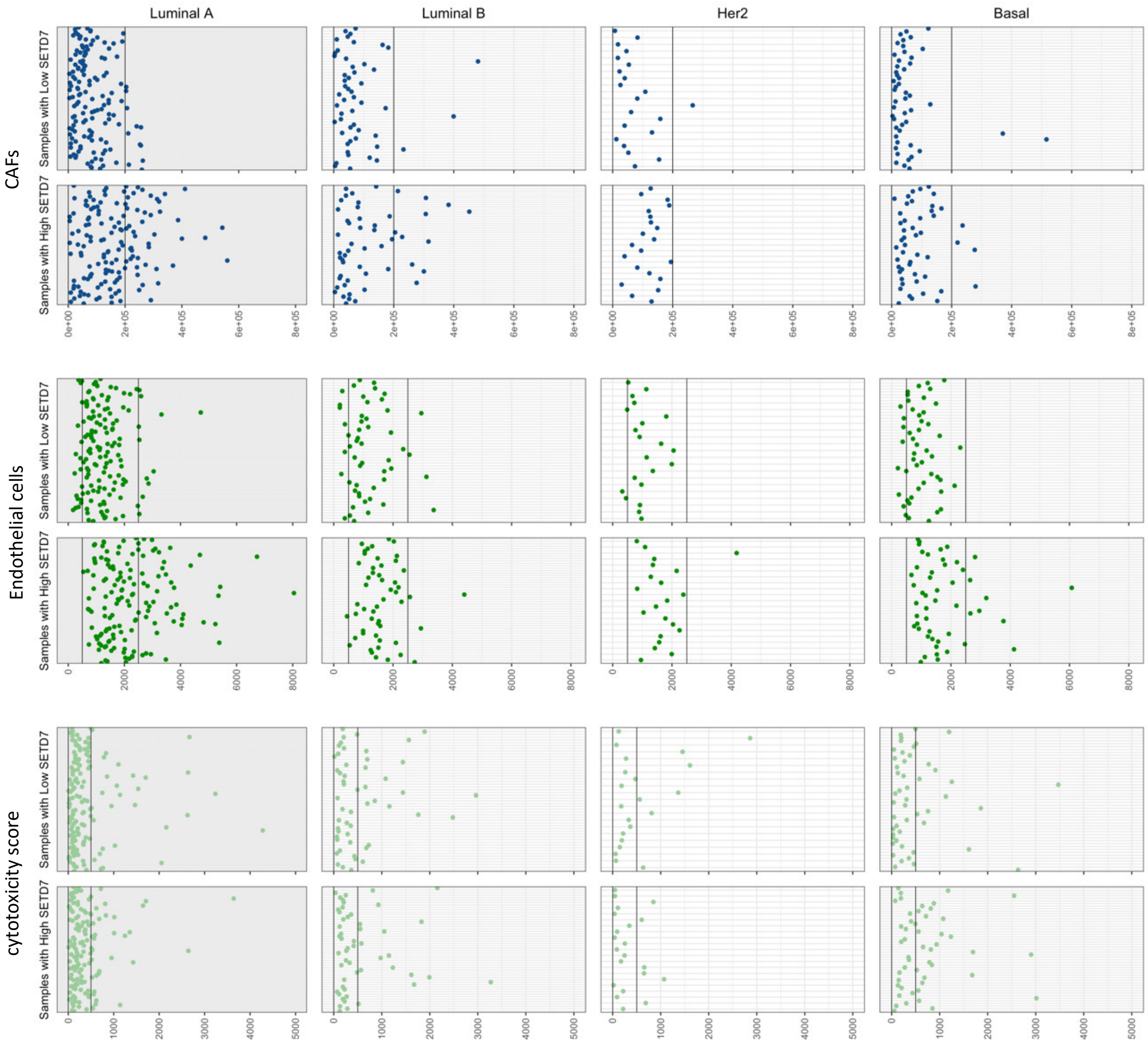

Figure S4. Predicted infiltration of cancer-associated fibroblasts (CAFs), endothelial cells and cytotoxicity score in samples with differential *SETD7* mRNA expression (high *vs* low) from different breast cancer PAM50 subtypes using mcp_count method from immunodeconv package and TCGA-BRCA data overall.

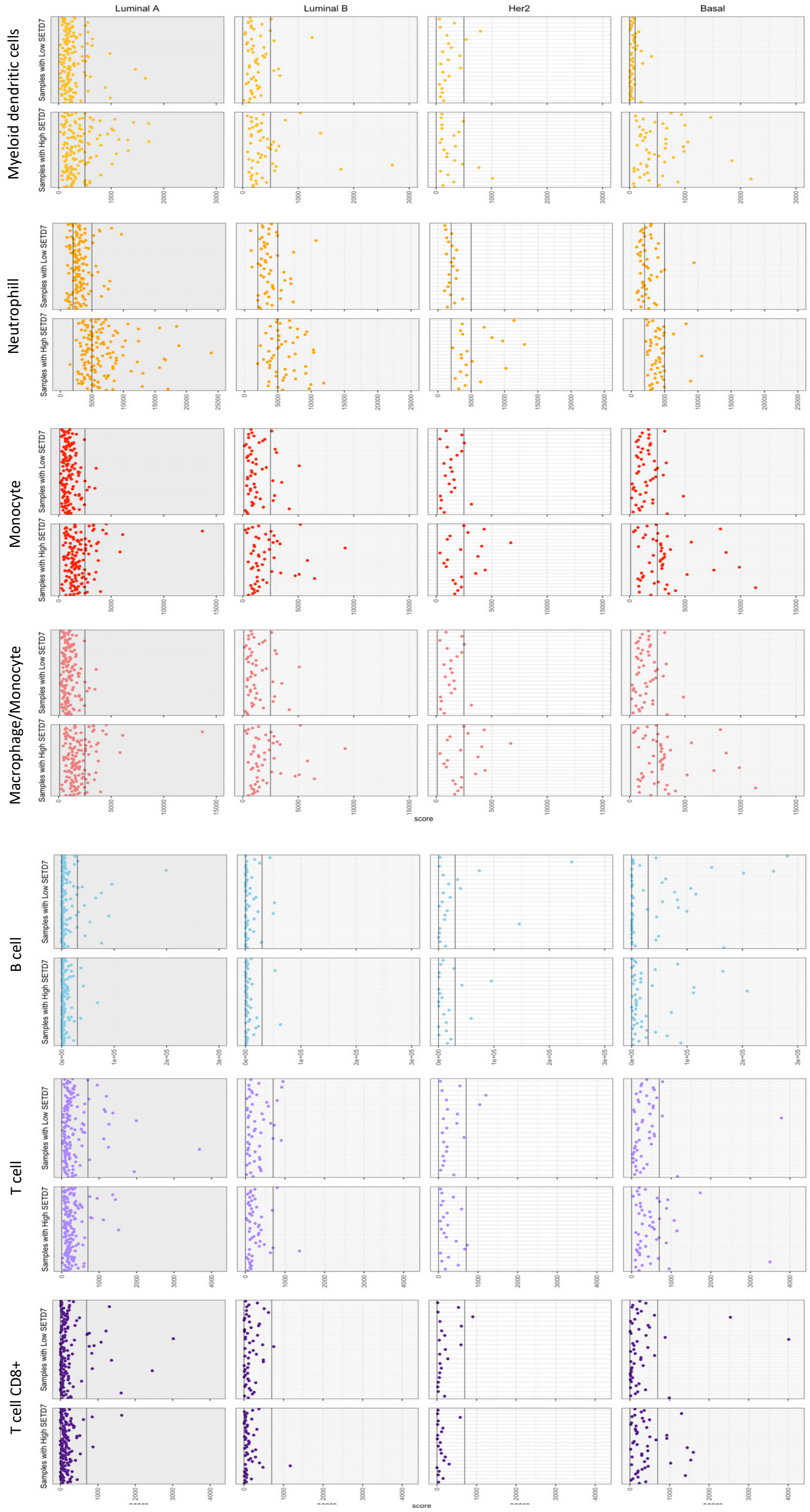

Figure S5. Predicted infiltration of immune cells in samples with differential *SETD7* mRNA expression (high *vs* low) from different breast cancer PAM50 subtypes, using mcp_count method from immunodeconv package and TCGA-BRCA data overall.

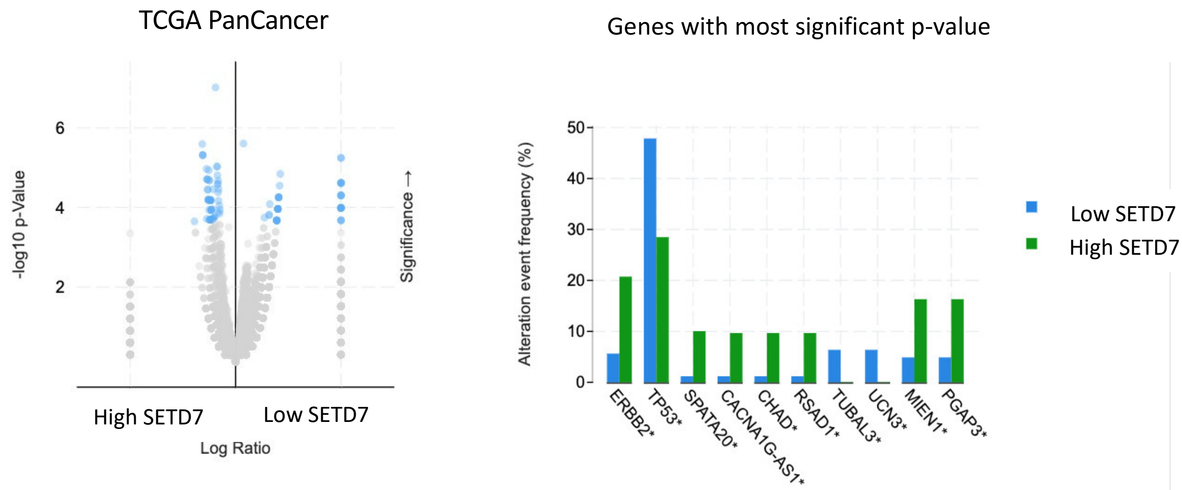

Figure S6. Genomic alterations associated *SETD7* mRNA differential expression from TCGA PanCancer Atlas in cBioPortal.

Retrieve TCGA-BRCA data from GDC

- n = 1102
- 56602 genes
- Clinical information (PAM50 subtype, tumour stage, vital status, followUp days)

SETD7 expression

(lower *vs* upper quartiles -> remove unchanged)

- Overall (552 / 1102 samples)
- Per subtype
  - Basal (98 / 194 samples)
  - Luminal A (284 / 567 samples)
  - Luminal B (104 / 207 samples)
  - HER2+ (42 / 82 samples)

Filter out genes that are low expressed

(FPKM < 1 in both High and Low SETD7 groups)

- Overall (56602 – 40950 = 15652 genes)
- Per subtype
  - Basal (15501 genes)
  - Luminal A (15632 genes)
  - Luminal B (14798 genes)
  - HER2+ (14735 genes)

Filter out genes that are not expressed in at least a quarter of the samples

(based on CPM values)

- Overall (15255 / 15652 genes)
- Per subtype
  - Basal (15044 / 15501 genes)
  - Luminal A (15097 / 15632 genes)
  - Luminal B (14634 / 14798 genes)
  - HER2+ (14548 / 14735 genes)

Differential expression

(Dseq2 pipeline, significant: padj<0.05 and |log2FC|>0.4)

- Overall (8325 / 15253 genes)
- Per subtype
  - Basal (6812 / 15044 genes)
  - Luminal A (7202 / 15097 genes)
  - Luminal B (6068 / 14634 genes)
  - HER2+ (5082 / 14548 genes)

(**a**)

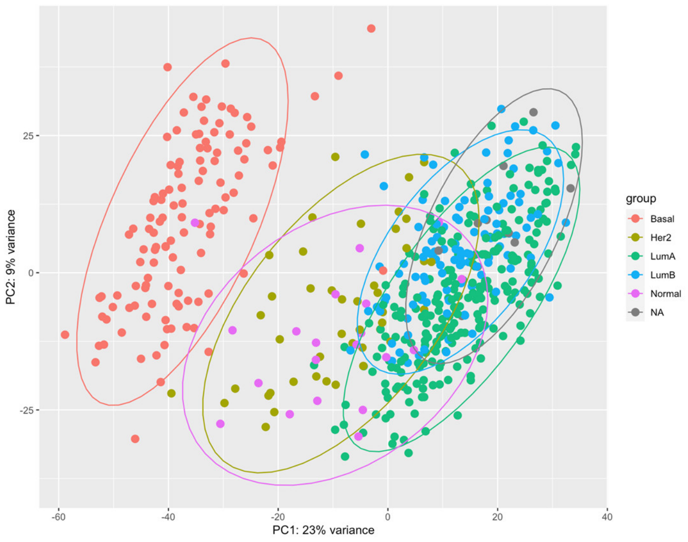

(**b**)

Figure S7. TCGA-BRCA data processing. (**a**) Scheme describing the TCGA-BRCA data processing; (**b**) PCA plot of TCGA-BRCA samples stratified by high- and low-SETD7.

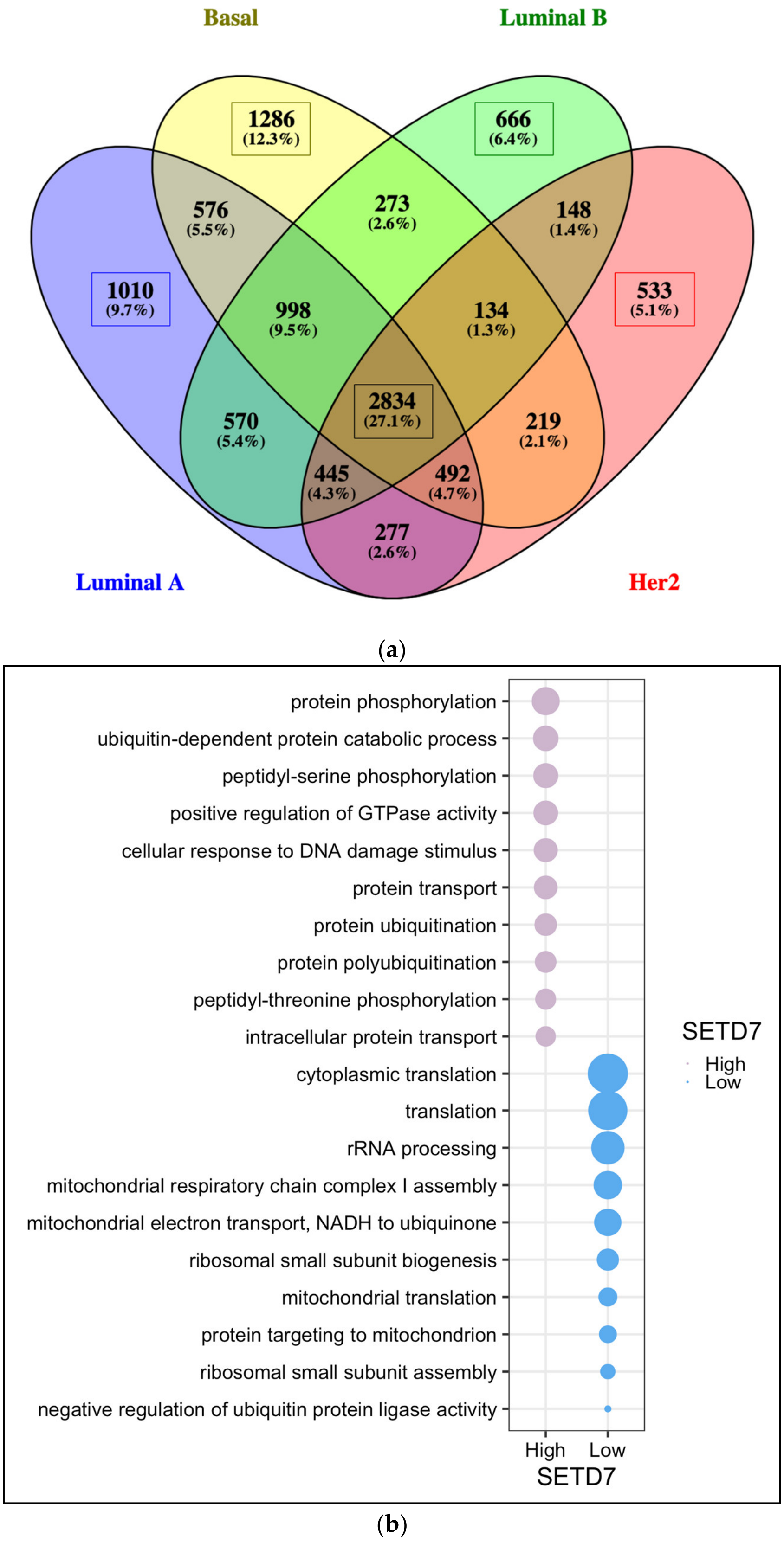

Figure S8. Analysis of genes associated with *SETD7* mRNA differential expression per subtype from TCGA-BRCA. (**a**) Venn diagram; (**b**) Top-10 Biological processes overrepresented by the genes common to all subtypes and significantly associates with *SETD7* differential expression (high and low distinctly).

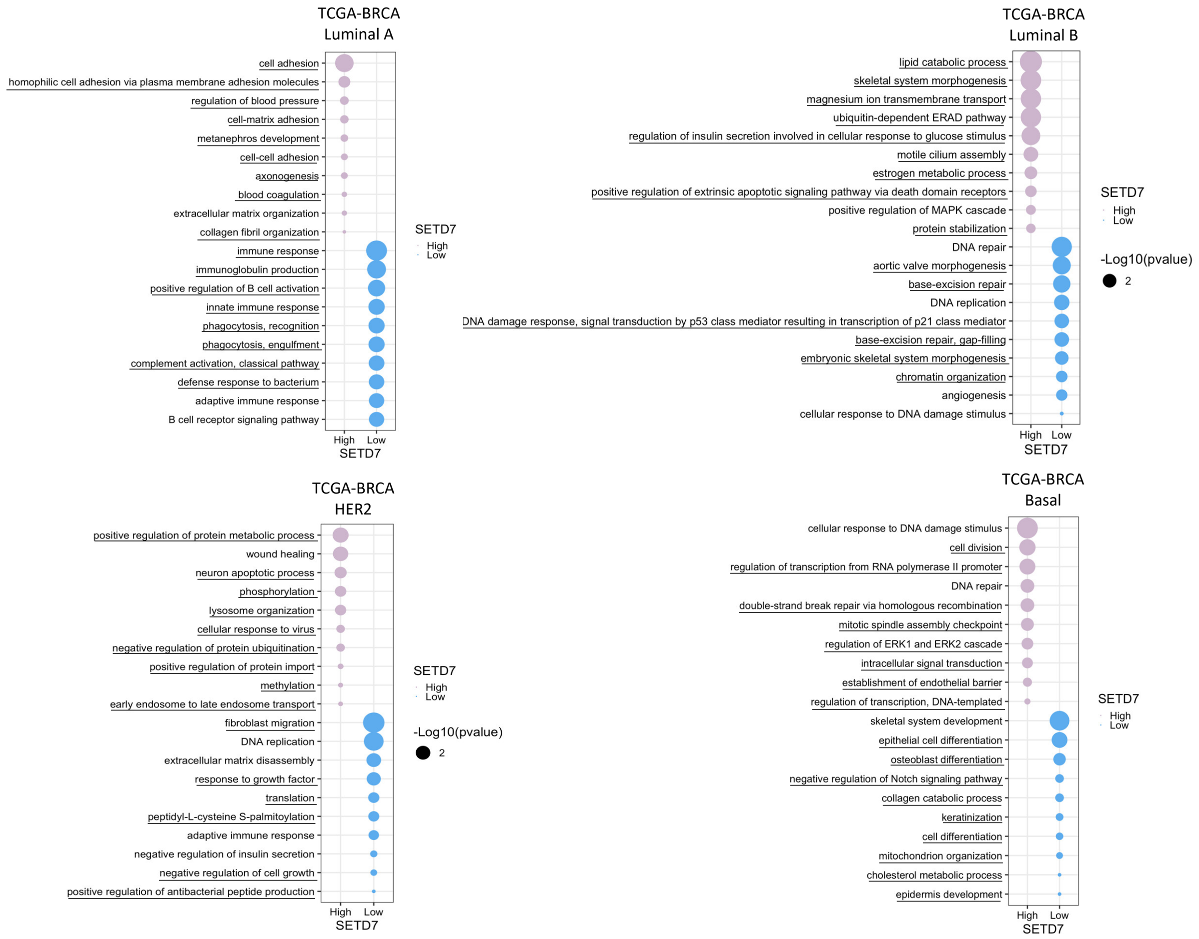

Figure S9. Top-10 biological processes overrepresented by the genes associated with *SETD7* differential expression (high and low distinctly) and unique for each subtype. Biological processes unique to each subtype are underlined.

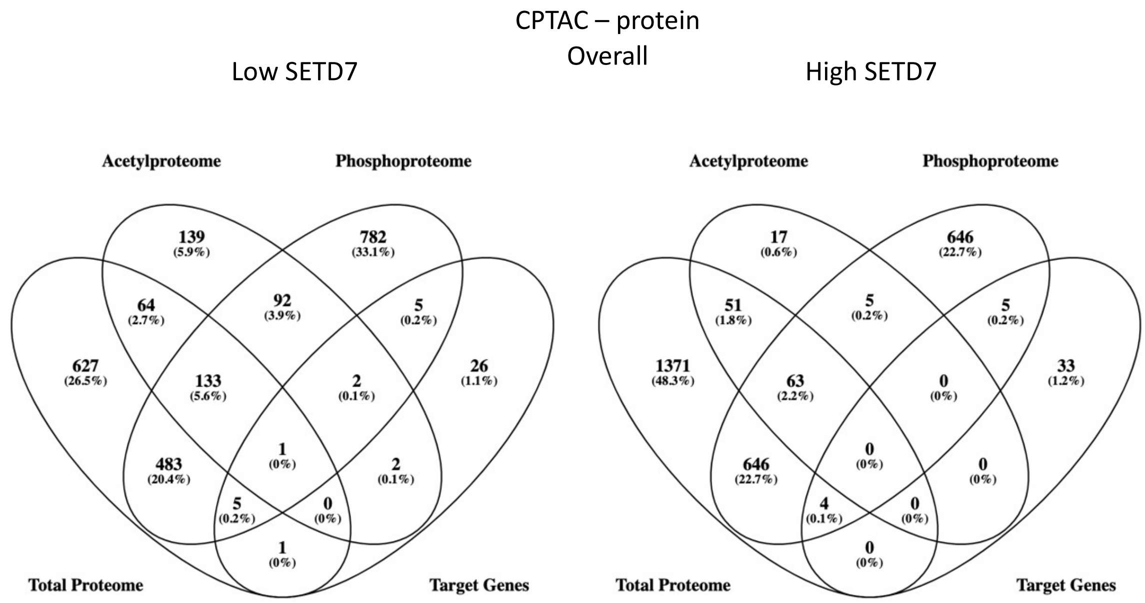

(a)

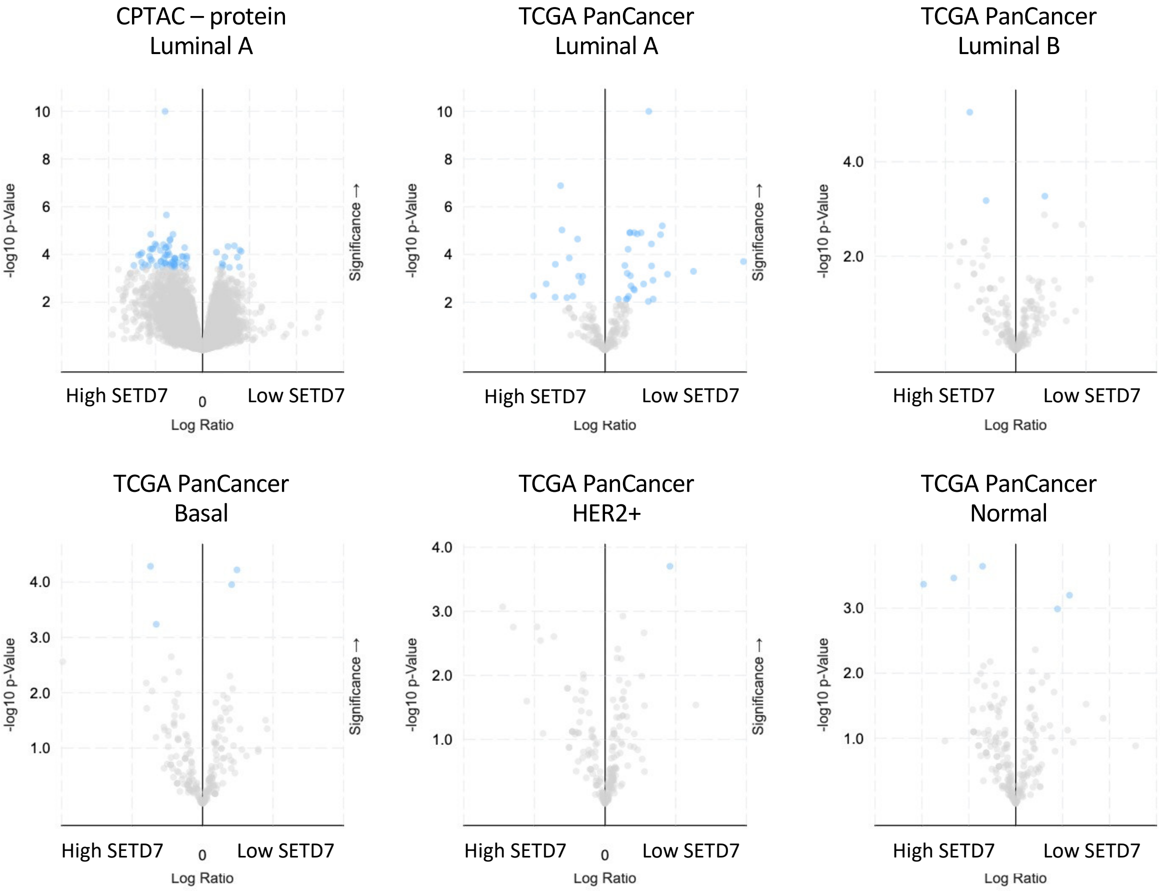

(b)

Figure S10. Analysis of proteins associated with SETD7 protein differential expression using CPTAC. (**a**) Venn diagrams showing the intersection of the results from total proteome, acetylproteome, phosphoproteome and target genes for high- and low-SETD7 groups distinctly. (**b**) Volcano plots of the total proteome analysed per subtype.
